# Genetic diversity of collaborative cross mice enables identification of novel rift valley fever virus encephalitis model

**DOI:** 10.1371/journal.ppat.1010649

**Published:** 2022-07-14

**Authors:** Haley N. Cartwright, Dominique J. Barbeau, Joshua D. Doyle, Ed Klein, Mark T. Heise, Martin T. Ferris, Anita K. McElroy

**Affiliations:** 1 University of Pittsburgh, School of Medicine, Department of Pediatrics, Division of Pediatric Infectious Disease, and Center for Vaccine Research, Pittsburgh, Pennsylvania, United States of America; 2 University of Pittsburgh, Division of Laboratory Animal Resources, Pittsburgh, Pennsylvania, United States of America; 3 Department of Genetics, University of North Carolina at Chapel Hill, Chapel Hill, North Carolina, United States of America; 4 Lineberger Comprehensive Cancer Center, University of North Carolina at Chapel Hill, Chapel Hill, North Carolina, United States of America; 5 Department of Microbiology and Immunology, University of North Carolina at Chapel Hill, Chapel Hill, North Carolina, United States of America; University of Pennsylvania, UNITED STATES

## Abstract

Rift Valley fever (RVF) is an arboviral disease of humans and livestock responsible for severe economic and human health impacts. In humans, RVF spans a variety of clinical manifestations, ranging from an acute flu-like illness to severe forms of disease, including late-onset encephalitis. The large variations in human RVF disease are inadequately represented by current murine models, which overwhelmingly die of early-onset hepatitis. Existing mouse models of RVF encephalitis are either immunosuppressed, display an inconsistent phenotype, or develop encephalitis only when challenged via intranasal or aerosol exposure. In this study, the genetically defined recombinant inbred mouse resource known as the Collaborative Cross (CC) was used to identify mice with additional RVF disease phenotypes when challenged via a peripheral foot-pad route to mimic mosquito-bite exposure. Wild-type Rift Valley fever virus (RVFV) challenge of 20 CC strains revealed three distinct disease phenotypes: early-onset hepatitis, mixed phenotype, and late-onset encephalitis. Strain CC057/Unc, with the most divergent phenotype, which died of late-onset encephalitis at a median of 11 days post-infection, is the first mouse strain to develop consistent encephalitis following peripheral challenge. CC057/Unc mice were directly compared to C57BL/6 mice, which uniformly succumb to hepatitis within 2–4 days of infection. Encephalitic disease in CC057/Unc mice was characterized by high viral RNA loads in brain tissue, accompanied by clearance of viral RNA from the periphery, low ALT levels, lymphopenia, and neutrophilia. In contrast, C57BL/6 mice succumbed from hepatitis at 3 days post-infection with high viral RNA loads in the liver, viremia, high ALT levels, lymphopenia, and thrombocytopenia. The identification of a strain of CC mice as an RVFV encephalitis model will allow for future investigation into the pathogenesis and treatment of RVF encephalitic disease and indicates that genetic background makes a major contribution to RVF disease variation.

## Author summary

Rift Valley fever virus (RVFV) is a negative sense RNA arbovirus of clinical relevance found throughout Africa and into the Middle East. In humans, RVF disease exhibits various clinical manifestations including acute febrile illness, hepatitis, hemorrhagic fever, and encephalitis. Unfortunately, existing murine models have been limited to studies of acute lethal hepatitis. Here, we identify a mouse model in which all animals survive acute hepatitis and go on to succumb to late onset encephalitis. A key feature of this new experimental system is administration of the virus in the footpad (to mimic mosquito bite), to model a natural route of infection. Importantly in this work, we used the Collaborative Cross recombinant inbred mouse resource to identify this RVFV CNS model. Therefore, our study demonstrates that host genetics can modulate RVF disease manifestations. The identification of this model also enables the field for high throughput, in vivo studies of central nervous system pathogenesis as well as in vivo evaluation of vaccines and therapeutics targeted against RVF encephalitis.

## Introduction

Rift Valley fever virus (RVFV) is a pathogen of both humans and livestock. In humans, Rift Valley fever (RVF) typically manifests as a self-limiting febrile illness. However, in up to 20% of cases people exhibit severe forms of disease including retinitis, hepatitis, hemorrhagic fever, or delayed-onset encephalitis [1–4]. Compared to the breadth of clinical outcomes in humans, inbred mouse strains challenged with wild-type (WT) RVFV are uniformly susceptible to infection and overwhelmingly develop a lethal acute hepatitis with occasional BALB/c mice surviving longer to display encephalitic manifestations [5–7]. There are no murine models that develop either hemorrhagic fever, retinitis, or uniform late-onset encephalitis. Pathogenesis and therapeutic studies for RVF disease manifestations besides hepatitis have therefore been restricted to larger animal models such as non-human primates (NHP), which are expensive and impractical for large throughput studies [8–10]. Young gerbils are also a model of RVF encephalitis, however, their susceptibility to disease wanes with age [11]. An alternative for the study of encephalitis in rodent models has been to administer virus directly into the nose or via aerosol, however, this does not represent the natural route of infection [12–15]. Thus, a murine model of encephalitis following peripheral RVFV exposure is needed.

The divergent RVFV clinical manifestations seen in humans have been associated with polymorphisms in innate immune signaling pathway molecules, suggesting that human clinical outcome is shaped by differences in the quality of the innate immune response [16]. Interestingly, unlike standard inbred mouse strains, rats have shown strain specific differences in disease susceptibility to RVFV. Distinct differences in severity of RVFV infection after peripheral challenge exist between Wistar-Furth (WF) and Lewis rats as well as differences in clinical manifestations between the acute hepatic WF rats and the late-onset encephalitic August-Copenhagen-Irish (ACI) rats [17–19]. These divergent clinical outcomes following RVFV infection across different rat strains and among humans suggest a genetic basis for disease variation. In the search for novel murine models of RVF disease, the genetically diverse Collaborative Cross (CC) resource was investigated in this study.

The CC is a genetically defined recombinant mouse panel derived from the systematic interbreeding of 8 founder strains representing >90% of all common genetic variation across *Mus musculus*: 5 classically used inbred mouse strains (A/J, C57BL/6J, 129S1/SvlmJ, NOD/ShiLtJ, NZO/HILtJ) and 3 wild-derived strains (CAST/EiJ, PWK/PhJ, WSB/EiJ) [20, 21]. These mouse crosses were then inbred for generations so that the resultant CC mouse strains were >90 percent homozygous and genetically defined while also containing a high level of genetic variation distributed randomly across each strain’s genome [22, 23]. With numerous mouse strains available for purchase, CC strain selection was based on the likelihood of each strain to exhibit increased resistance to infection. The interferon-induced GTPase, MxA, has been shown to inhibit RVFV replication in vitro, therefore its mouse homologue Mx1 was used as the selection criteria for this study [24]. Twenty CC mouse strains that are known to have a functional wild-derived Mx1 locus were selected for challenge with RVFV [25]. These 20 CC strains were evaluated for their susceptibility to RVFV infection and characterized through clinical, virologic, immunologic, hematologic, and metabolic readouts. A direct comparison of C57BL/6 mice and the CC strain that had the most divergent clinical outcome was used to define differences in disease manifestation and progression over time. This report provides the groundwork for the use of the CC Resource in defining the genetic basis of RVF disease phenotype and details the identification of the CC057 strain as a murine model of RVF encephalitis.

## Results

### CC genetic diversity drives divergent RVF disease manifestations

**Fig 1:**
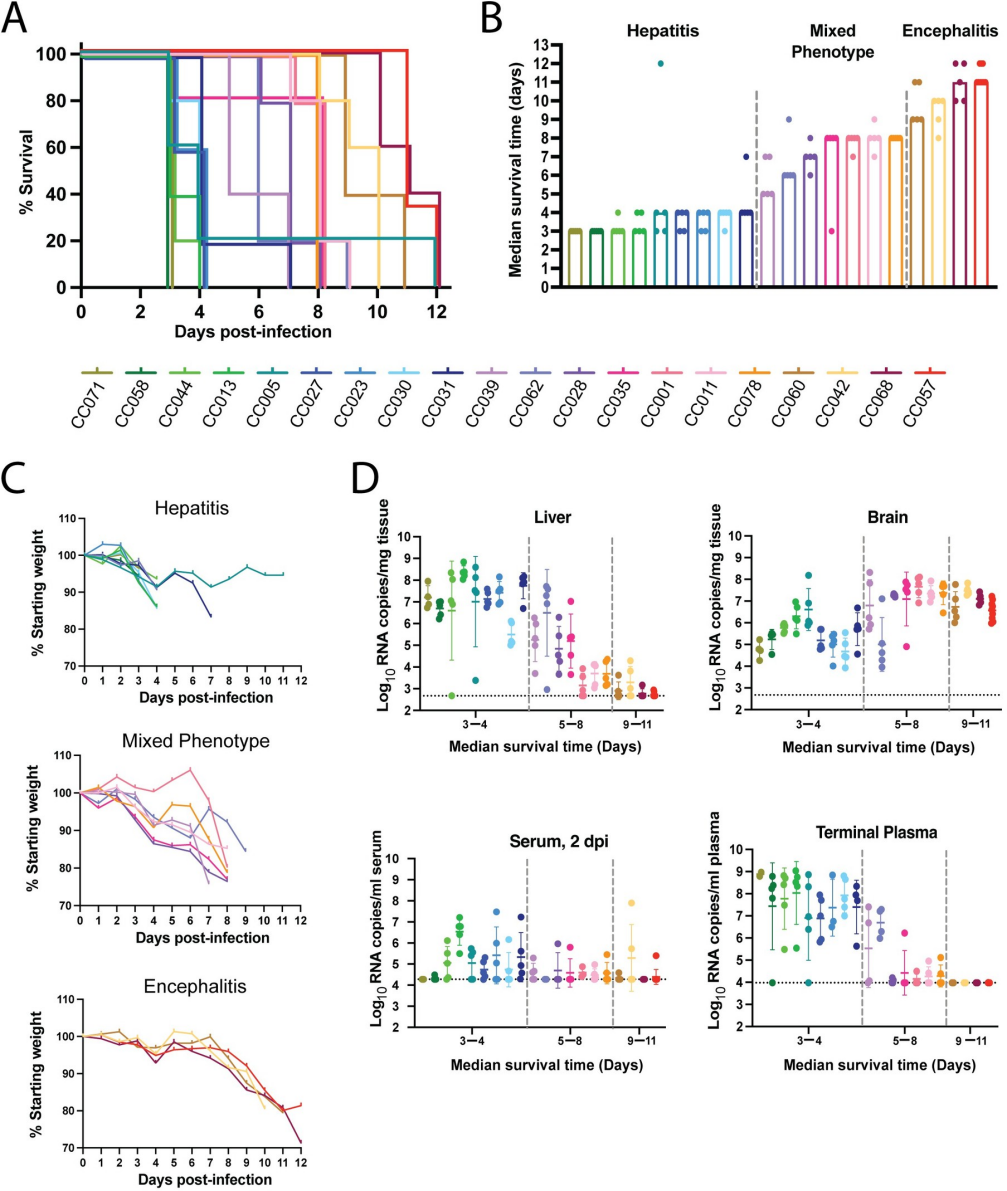
Disease phenotypes identified after RVFV infection in CC strains. A) Survival curve of 20 different CC mouse strains following RVFV infection. Each line represents 5 female mice apart from CC057 mice (n = 9, 5 female and 4 male). B) Median time to death across CC strains. C) Mean percent of starting body weight over the course of infection with CC strains split into three categories of disease. D) qRT-PCR based assessment of viral RNA loads in serum at 2 dpi and from tissues and plasma at time of euthanasia. CC strains are separated into three categories of disease by two dashed vertical grey lines. Serum and plasma viral loads were assessed if sufficient sample was present (n≤5 per CC strain) with data shown as geometric mean ± geometric SD. LOD of assays noted by dotted horizontal line.

To evaluate RVF disease phenotypes in genetically diverse mouse strains, 20 CC strains were challenged with 2 TCID_50_ of WT ZH501 RVFV via footpad (FP) injection. All mice universally succumbed to this challenge dose, however, strains varied widely in their time to death (Fig 1A and 1B). Three categories of RVF disease were identified based on gross pathology, clinical symptoms, and median time to death: hepatitis, mixed phenotype, and encephalitis (Fig 1). Strains classified as hepatic died early in infection; median of 3–4 days post-infection (dpi), within the timeframe of known inbred mouse models of RVF hepatitis. These mice had grossly enlarged livers and experienced rapid decline in weight immediately before euthanasia criteria were met (Fig 1C). In contrast, strains classified as encephalitic died consistently late in infection; median of 9–11 dpi, showing progressive weight loss late in the disease course preceding euthanasia. Clinical symptoms in these strains included lateral eye deviation, circling, ataxia, seizure, and hind limb paralysis. These strains lacked gross liver pathology at time of death. Finally, strains classified as mixed phenotype, displayed a gradient of hepatic and encephalitic disease symptoms, and met euthanasia criteria between a median of 5–8 dpi.

These three classifications of RVF disease phenotype were supported by distinct viral RNA load patterns within key tissues. Hepatic-classified mice had the highest viral RNA loads in the liver at the time of death while encephalitic mice died with minimal liver viral RNA loads but high brain viral RNA loads (Fig 1D). In addition to having high viral RNA loads in the liver, hepatic mice were viremic with high viral RNA loads also present in the serum and other tissues (Figs 1D and S1) at the time of death. In contrast, mice dying of late-onset encephalitis succumbed to disease despite near clearance of viral RNA from peripheral tissues and the blood.

**Fig 2:**
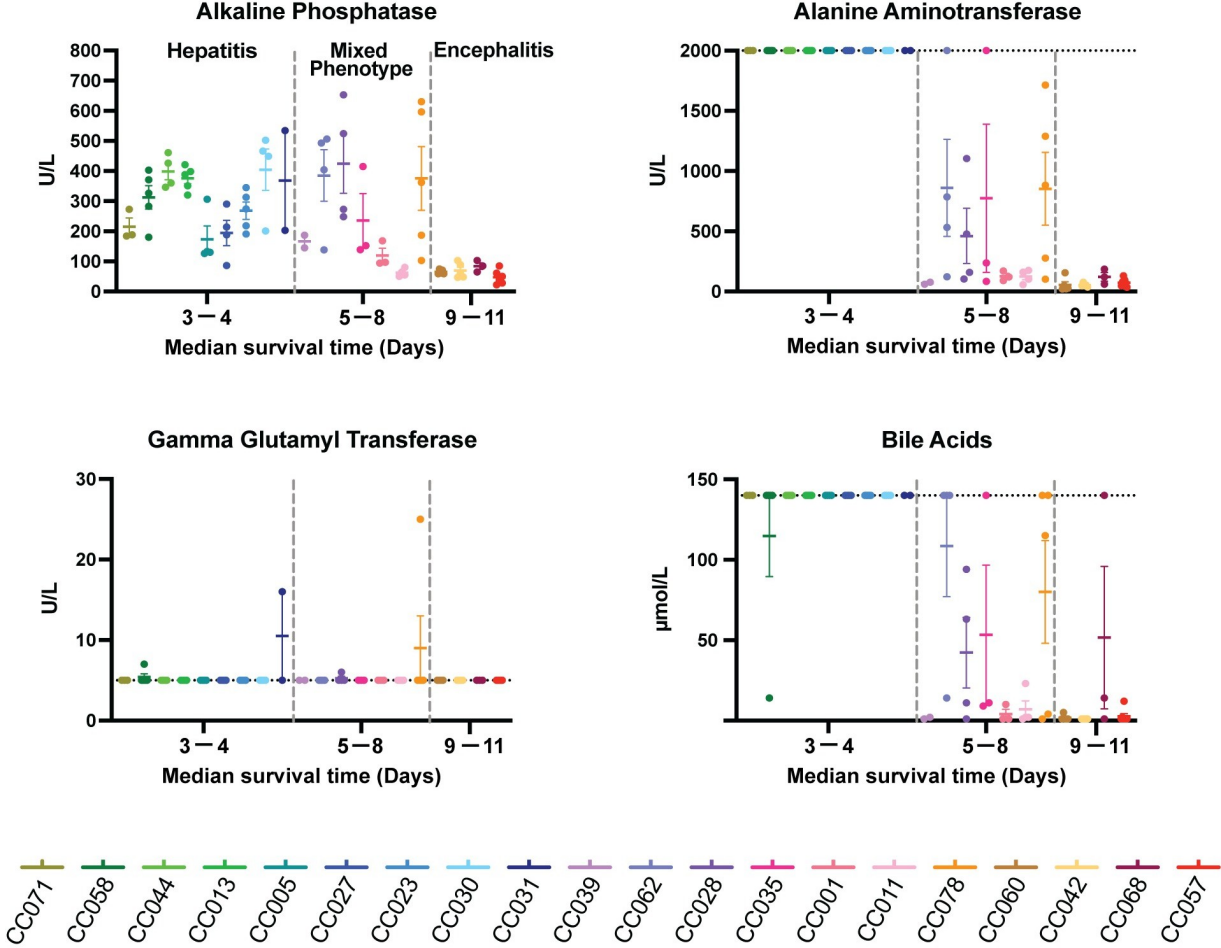
Liver chemistry profiles vary across CC strains and correlate with RVF disease. CC strains are separated into three categories of disease by two vertical dashed grey lines. CHEM was run if sufficient sample was present (n≤5 per CC strain). Data shown as mean ± SD. If data were outside the LOD, the upper or lower LOD for assays are noted by horizontal dotted lines. Alanine Aminotransferase upper LOD: 2000 U/L; Gamma Glutamyl Transferase lower LOD: 5 U/L; Bile Acids upper LOD: 140 μmol/L.

In addition to large differences in viral RNA distribution and load between hepatic and encephalitic mice, these divergent disease phenotypes displayed clinical differences in blood chemistry (CHEM) data at time of death (Fig 2). Mice that died early of hepatitis had elevated alkaline phosphatase (ALP), alanine aminotransferase (ALT), bile acids, while encephalitic mice died in the absence of clinical markers of liver involvement. Notably, gamma glutamyltransferase (GGT), a marker of biliary disease, was not particularly elevated in mice with hepatitis arguing against involvement of the biliary tree. Other serum chemistry markers and complete blood counts (CBC) were not consistently different between the phenotypes at the time of euthanasia (S2 Fig).

To characterize the humoral response to infection, all mice that succumbed later than 5 dpi were assessed by RVFV-specific enzyme-linked immunosorbent assay (ELISA). Those strains found to be positive for RVFV-specific antibodies were further assessed by focus reduction neutralization test (FRNT). Regardless of CC strain, mice mounted an antibody response starting around 8 dpi (S3 Fig). Mice that survived longer post-infection had a more robust neutralizing antibody response than those that died earlier. However, even mice with the strongest neutralizing antibody response at their time of death succumbed to RVF encephalitic disease.

### CC057 mice have a unique disease course compared to C57BL/6 mice

**Fig 3:**
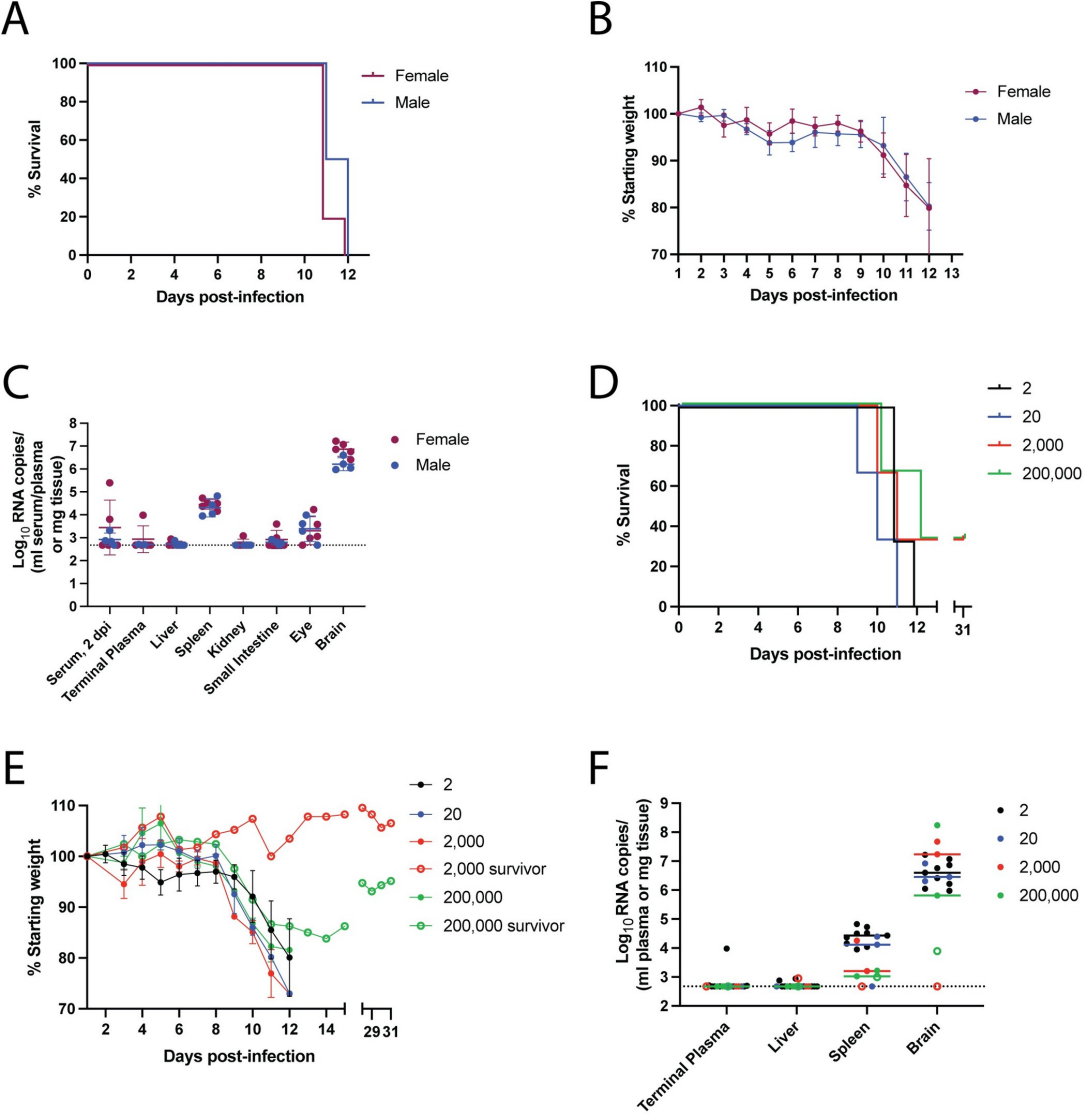
The RVF clinical phenotype in CC057 mice is not sex-dependent and was observed at increasing challenge doses. A) Survival curve of CC057 mice split by sex (female n = 5, male n = 4). B) Percent of starting body weight over the course of infection. Data presented as mean ± SD. C) qRT-PCR based assessment of viral RNA loads in CC057 serum at 2 dpi and tissues and plasma at time of euthanasia. Data previously presented in Figs 1 and S1, now split by sex. Data shown as geometric mean ± geometric SD. D) Survival curve of CC057 mice after challenge with different RVFV doses (2 TCID_50_ n = 9; 20 TCID_50_ n = 3; 2,000 TCID_50_ n = 3; 200,000 TCID_50_ n = 3). E) Percent of starting body weight over the course of infection after RVFV challenge at different doses. Survivor weights not plotted between 16 dpi and 27 dpi. Data presented as mean ± SD. F) qRT-PCR based assessment of viral RNA loads in CC057 tissues and plasma at time of euthanasia after RVFV challenge at different doses (2 TCID_50_ n = 9; 20 TCID_50_ n = 3; 2,000 TCID_50_ n = 3; 200,000 TCID_50_ n = 3). Data shown as geometric mean ± geometric SD. Survivor mice viral RNA titers are represented as open circles. 2 TCID_50_ challenge dose data in panels D-F are the same 9 mice as presented before in panels A-C. LOD for tissue samples noted by dotted line at 473 RNA copies. LOD for serum, 2dpi samples = 18,960 RNA copies. LOD for terminal plasma samples = 9,480 RNA copies.

From the four CC strains that developed consistent late-onset encephalitic disease, the CC057 strain was selected for additional in-depth studies. The RVFV encephalitic phenotype was found to be sex-independent in the CC057 mouse strain, with both female and male mice displaying nearly identical survival curves and weight loss trends (Fig 3A and 3B). CC057 viral RNA data revealed uniformity between sexes with viral RNA loads being consistently highest in the brain regardless of sex (Fig 3C). To solidify the CC057 strain as a model of RVF encephalitis regardless of challenge dose, CC057 mice were infected with additional doses of RVFV. Irrespective of challenge dose the late-onset RVF encephalitis phenotype was observed with similar survival curves and weight loss trends (Fig 3D and 3E). Interestingly, one mouse in both of the two highest challenge doses survived RVFV infection. These two mice were confirmed to be true survivors with anti-RVFV ELISA titers >24,300 at the experimental endpoint of 31 dpi. All mice that succumbed to RVF, across all challenge doses, had the highest viral RNA loads in the brain with close to undetectable levels in the liver (Fig 3F).

**Fig 4:**
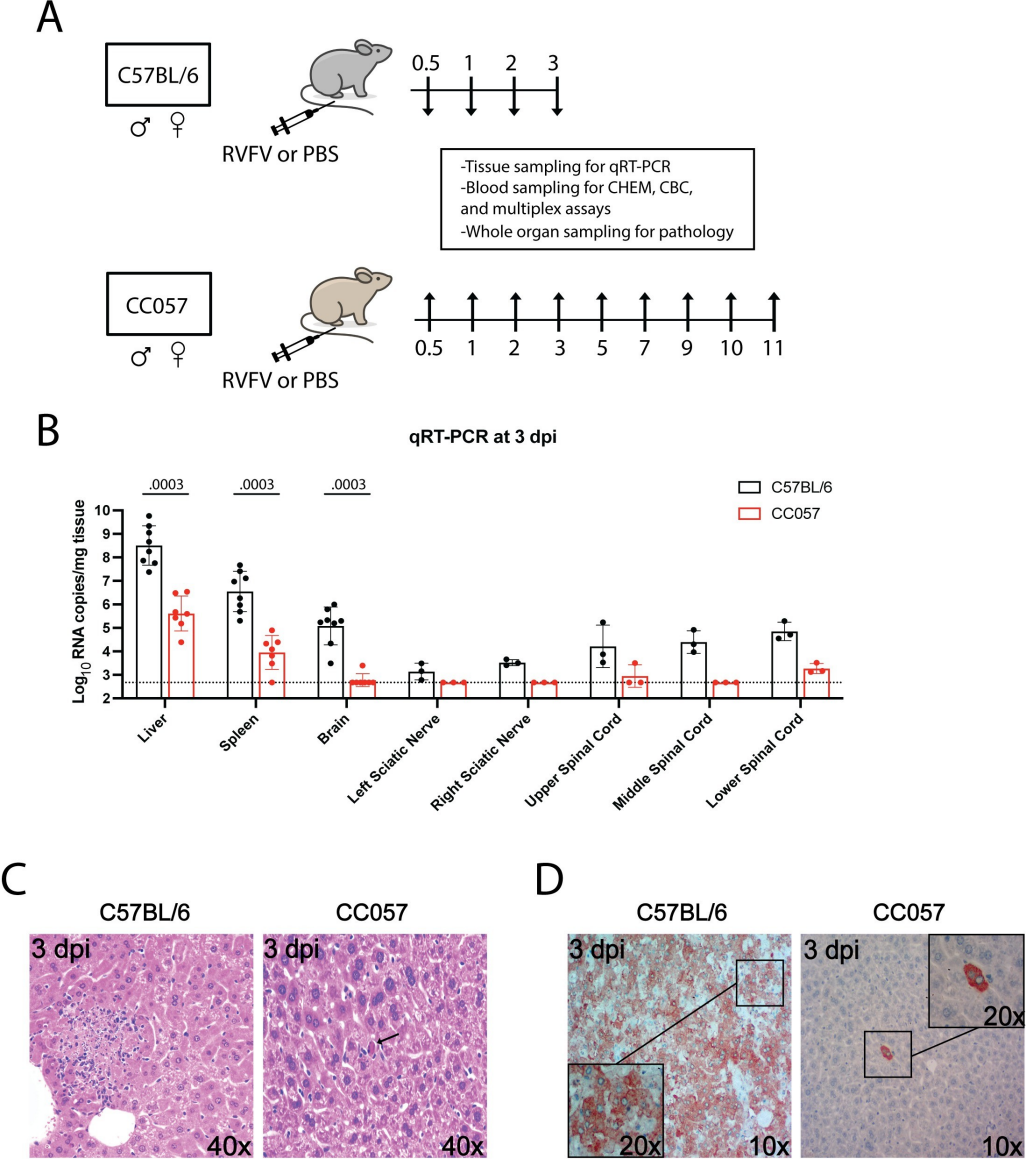
CC057 mice have self-limiting viral replication and spread in the liver. A) Experimental design: at each timepoint, tissue and blood samples were taken to assess virologic, immunologic, hematologic, and histologic readouts. B) qRT-PCR based assessment of viral RNA loads in tissues of C57BL/6 and CC057 mice at 3 dpi. Data shown as geometric mean ± geometric SD (n = 3–8 mice/tissue type). Each data point shown represents an individual mouse. LOD of assay noted by dotted line. Comparison of viral RNA load between mouse strains was performed in each tissue by Mann-Whitney (liver p = 0.0003; spleen p = 0.0003; brain p = 0.0003). C) Representative H&E-stained sections of formalin-fixed paraffin-embedded livers at 3 dpi. Arrow points to focal individual cell degeneration (CC057). D) IHC of RVFV antigen (brown) in representative sections of formalin-fixed paraffin-embedded livers at 3 dpi.

To directly compare hepatic and encephalitic RVF disease progression, the classically used C57BL/6 hepatitis model and the newly identified CC057 encephalitis model were infected with 2 TCID_50_ WT ZH501 RVFV in the left footpad and serially euthanized at various timepoints (Fig 4A). At each timepoint, tissue and blood samples were taken to assess virologic, hematologic, metabolic, histologic, and immunologic readouts. Apart from one viral RNA positive C57BL/6 liver sample at 2 dpi, all other C57BL/6 and CC057 mouse tissues were below the limit of detection (LOD) for viral RNA at 0.5, 1, and 2 dpi. In contrast, at 3 dpi, viral RNA was detectable in all sampled tissues in C57BL/6 mice with the highest loads being present in the liver (Fig 4B). Viral RNA was also present in various CC057 tissues including the liver; however, C57BL/6 viral RNA loads in the liver, spleen, and brain were significantly higher than those in CC057 mice.

No definitive virus-induced cytopathology was seen in either C57BL/6 or CC057 livers before 3 dpi. At 3 dpi, lesions appeared in the livers of all mice; for both C57BL/6 and CC057 mice the most common findings were foci of hepatocellular degeneration and necrosis. These were generally associated with inflammatory, often neutrophilic, infiltrates. Livers varied widely in frequency of necrotic foci, from substantial in C57BL/6 livers to more infrequent focal individual cell degeneration in CC057 livers (Fig 4C). Differences in the extent of hepatocellular damage were paralleled by the large difference in viral antigen staining seen by immunohistochemistry (IHC) between the two mouse strains. C57BL/6 mice appropriately had high levels of antigen staining in the liver at 3 dpi (3 of 3 mice) with one mouse staining positive as early as 2 dpi (Fig 4D). Contrastingly, at 3 dpi the extent of antigen staining in CC057 livers was considerably lower with only sporadic single hepatocytes staining positive (3 of 3 mice).

**Fig 5:**
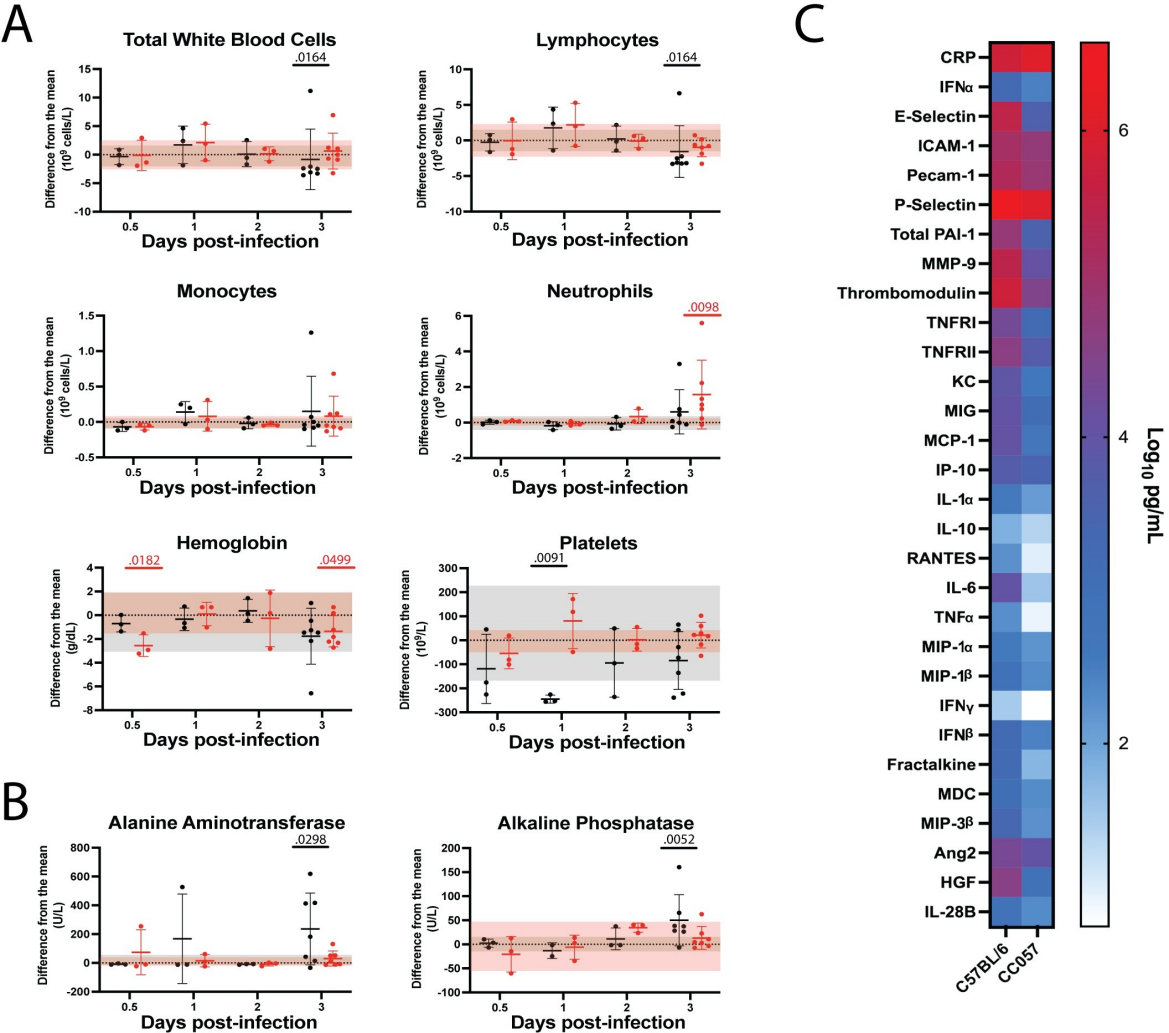
Acute phase blood biomarkers in C57BL/6 versus CC057 mice. A) CBC and B) CHEM over time with data presented as a function of difference from the uninfected mean, shown as mean ± SD (n = 3–7 mice/time point). Each data point shown represents an individual mouse. The uninfected means and normal ranges for C57BL/6 and CC057 mice were determined by performing CBC and CHEM on blood from 9 uninfected C57BL/6 mice and 9 uninfected CC057 mice. Uninfected normal ranges from the mean for C57BL/6 and CC057 mice are represented by grey and pink horizontal shading respectively. Comparisons of CBC data were performed at each timepoint by Mann-Whitney to compare RVFV-infected samples to uninfected control samples for each mouse strain separately (C57BL/6: Total white blood cells 3 dpi p = 0.0164; Lymphocytes 3 dpi p = 0.0164; Platelets 1 dpi p = 0.0091. CC057: Neutrophils 3 dpi p = 0.0098; Hemoglobin 0.5 dpi p = 0.0182, 3 dpi p = 0.0499). Comparisons of CHEM data were performed at each timepoint by Mann-Whitney to compare RVFV-infected samples to uninfected control samples for each mouse strain separately (C57BL/6: ALT 3 dpi p = 0.0298; ALP 3 dpi p = 0.0052). C) Analyte levels in the plasma at 3 dpi. Data shown as the mean (C57BL/6 n = 15; CC057 n = 3).

CBC and CHEM data revealed another critical aspect to the divergence in disease course between hepatic and encephalitic RVF. White blood cell counts did not vary significantly from baseline in either C57BL/6 or CC057 mice before 3 dpi (Fig 5A). At 3 dpi, however, clear differences emerged with C57BL/6 mice alone exhibiting significant leukopenia and lymphopenia. Although some C57BL/6 mice had elevated levels of neutrophils at 3 dpi, CC057 mice displayed significantly higher neutrophilia from baseline at this critical point in infection. Interestingly, only CC057 mice had anemia at 0.5 and 3 dpi while C57BL/6 mice alone exhibited thrombocytopenia. Clinical markers of liver dysfunction, ALT and ALP, were significantly elevated in the hepatic C57BL/6 mouse model at 3 dpi (Fig 5B). CHEM data with no physiologically relevant changes from baseline for either C57BL/6 or CC057 mice are included in the supplementary material (S4 Fig).

To further compare hepatic and encephalitic disease courses, the concentrations of 32 analytes were measured in the plasma of C57BL/6 and CC057 mice over the course of infection using multiplex immune assays. Selected analytes included markers of inflammation and markers of endothelial or barrier function. Baseline levels of 11 analytes were found to be significantly lower in CC057 compared to C57BL/6 mice, suggesting a slightly lower baseline inflammatory environment in the CC057 mouse strain (S5 Fig). With the goal of identifying early biomarkers of hepatic versus encephalitic disease outcome, analyte levels at 0.5, 1, and 2 dpi were compared between C57BL/6 and CC057 mice by two-way ANOVA. Unfortunately, of the 32 measured analytes no significant biomarkers of outcome were found at these early timepoints with 3 mice per strain analyzed per timepoint. However, at the critical 3 dpi timepoint when most C57BL/6 mice succumbed to hepatitis, nearly all measured C57BL/6 analyte levels were highly elevated including IL-6 and HGF, two inflammatory markers associated with the liver (Fig 5C). To compare analyte levels between C57BL/6 and CC057 mice, data were normalized to their respective uninfected mean (Table 1). By Mann-Whitney, 21 of 32 analyte levels were significantly higher in C57BL/6 mice as compared to CC057 mice (Table 1). Multiplex data were excluded for CD40L and IL-9 at 3 dpi due to a lack of data points above the limit of detection for either C57BL/6 or CC057 mice.

**Table 1:** C57BL/6 and CC057 analyte mean and 95% Confidence Interval (CI) at 3 dpi as a function of difference from the uninfected mean.

Analyte (pg/mL)	Mean C57BL/6 difference from uninfected	C57BL/6 95% CI	Mean CC057 difference from uninfected	CC057 95% CI	Significance by Mann-Whitney (C57BL/6 versus CC057)
CRP	-61,633,583	[-80,746,132, -42,521,035]	-9,429,167	[-60,072,281, 41,213,948]	P = 0.0270
IFN-α	2,621	[587, 4,656]	283	[-351, 916]	ns
E-Selectin	303,293	[216,118, 390,467]	-4,646	[-101,714, 92,422]	P = 0.0257
ICAM-1	59,309	[33,804, 84,814]	12,759	[-18,375, 43,892]	ns
Pecam-1	132,361	[76,664, 188,059]	-1,357	[-56,773, 54,059]	ns
P-Selectin	4,667,448	[-63,667, 9,398,563]	166,311	[-874,149, 1,206,772]	P = 0.0098
Total PAI-1	83,365	[54,090, 112,640]	3,236	[-860, 7,332]	P = 0.0025
MMP-9	344,725	[242,434, 447,015]	9,459	[-10,451, 29,369]	ns
Thrombomodulin	759,107	[441,391, 1,076,822]	2,588	[-14,486, 19,662]	P = 0.0270
TNFRI	23,949	[11,392, 36,506]	493	[-288, 1,274]	P = 0.0172
TNFRII	49,081	[30,699, 67,464]	3,476	[-4,250, 11,203]	P = 0.0172
KC	11,823	[7,659, 15,987]	188	[-12, 388]	P = 0.0012
MIG	12,486	[8,424, 16,548]	1,286	[15, 2,557]	P = 0.0049
MCP-1	14,664	[10,604, 18,725]	511	[-218, 1,240]	P = 0.0086
IP-10	5,742	[3,577, 7,906]	2,800	[1,650, 3,950]	P = 0.0392
IL-1α	-882	[-974, -791]	26	[-75, 128]	P = 0.0025
IL-10	75	[53, 97]	19	[6, 32]	P = 0.0098
RANTES	165	[118, 211]	-1	[-13, 12]	P = 0.0098
IL-6	16,131	[11,722, 20,540]	46	[-46, 137]	P = 0.0012
TNFα	256	[169, 343]	2	[-9, 14]	P = 0.0049
MIP-1α	216	[155, 277]	-16	[-16, -16]	P = 0.0025
MIP-1β	1,077	[685, 1,469]	182	[-34, 398]	ns
IFN-γ	30	[20, 41]	-1 (undetectable)	[-1, -1]	N/A
IFN-β	3,057	[265, 5,849]	220	[52, 388]	P = 0.0049
Fractalkine	1,518	[897, 2,138]	-24	[-83, 36]	ns
MDC	1,106	[385, 1,826]	61	[-106, 227]	P = 0.0172
MIP-3β	2,694	[1,881, 3,508]	-19	[-223, 185]	P = 0.0098
Ang2	16,248	[10,387, 22,110]	649	[-6,846, 8,144]	P = 0.0159
HGF	49,599	[36,548, 62,650]	-272	[-525, -19]	P = 0.0184
IL-28B	482	[265, 700]	-28	[-334, 278]	ns

### CC057 mice are a novel model of RVF encephalitic disease

**Fig 6:**
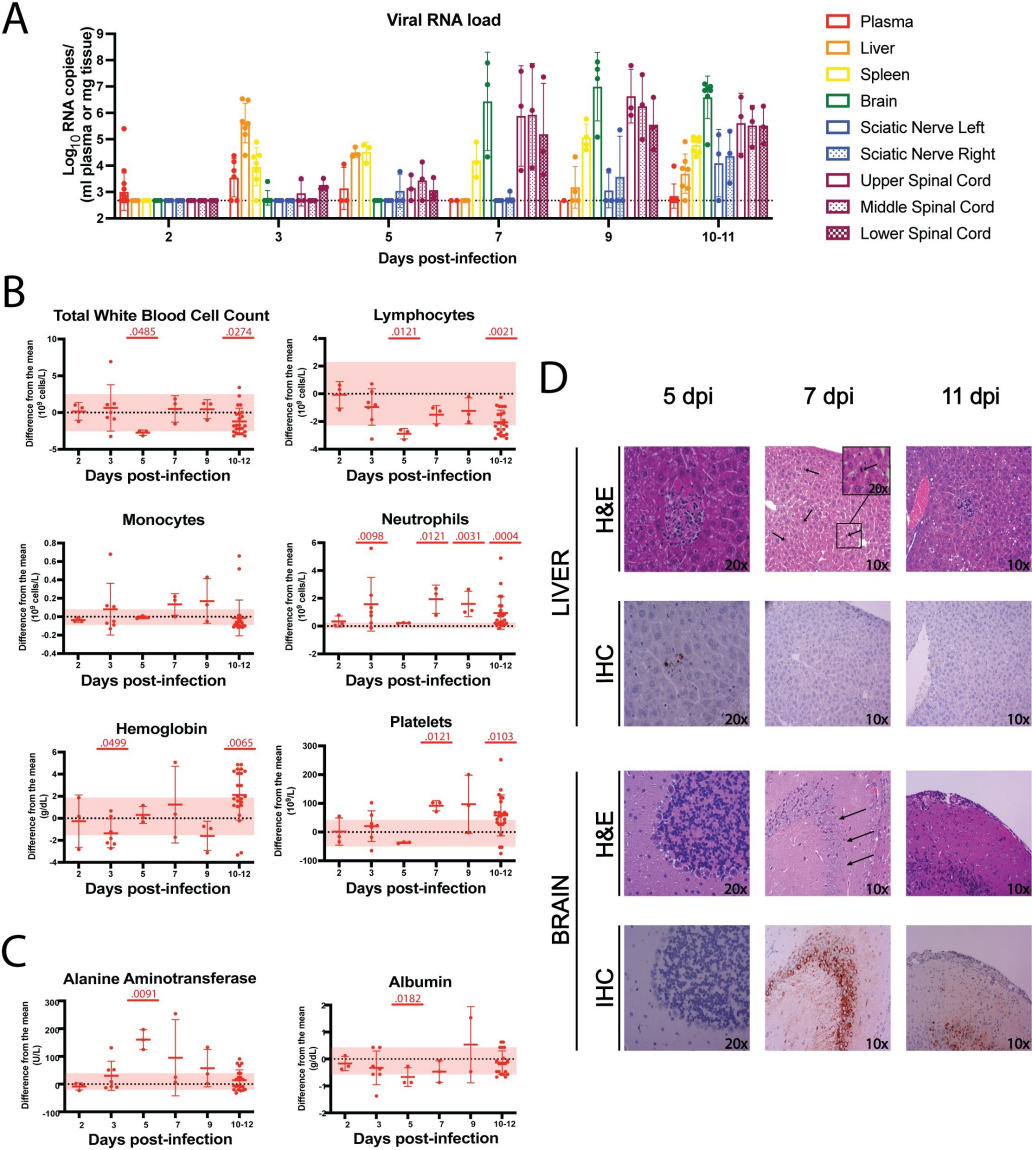
CC057 mice progress on to late-onset encephalitis after a self-limited hepatitis. A) qRT-PCR assessment of viral RNA loads in CC057 plasma and tissues over time. Data shown as geometric mean ± geometric SD (N = 1-16/tissue type). LOD for tissue samples noted by dotted line at 473 RNA copies. LOD for plasma samples = 18,960 RNA copies. B) CBC and C) CHEM over time with data presented as a function of difference from the uninfected mean, shown as mean ± SD (n = 3–23 mice/time point). Each data point shown represents an individual mouse. The uninfected means and normal ranges for CC057 mice were determined by performing CBC and CHEM on blood from 9 uninfected CC057 mice. 2 and 3 dpi data previously presented in Fig 5A and 5B. Pink horizontal shading represents uninfected normal ranges from the mean for CC057 mice. Comparisons of CBC data were performed at each timepoint by Mann-Whitney to compare RVFV-infected samples to uninfected control samples (Total white blood cells 5 dpi p = 0.0485, 10–12 dpi p = 0.0274; Lymphocytes 5 dpi p = 0.0121, 10–12 dpi p = 0.0021; Neutrophils 3 dpi p = 0.0098, 7 dpi p = 0.0121, 9 dpi p = 0.0031, 10–12 dpi p = 0.0004; Hemoglobin 3 dpi p = 0.0499, 10–12 dpi p = 0.0065; Platelets 7 dpi p = 0.0121, 10–12 dpi p = 0.0103). Comparisons of CHEM data were performed at each timepoint by Mann-Whitney to compare RVFV-infected samples to uninfected control samples (ALT 5 dpi p = 0.0091; Albumin 5 dpi p = 0.0182). D) Representative H&E-stained sections of formalin-fixed paraffin-embedded livers and brains from RVFV-infected CC057 mice at different times post-infection. Liver arrows point to active hepatocellular mitotic events while brain arrows point to neuronal dropout. IHC of RVFV antigen (brown) in representative sections of formalin-fixed paraffin-embedded RVFV-infected livers and brains at different times post-infection. The presented brain images are from different regions of the brain: cerebellum (5 dpi), hippocampus (7 dpi), and cortex (11 dpi).

To characterize disease course in the CC057 encephalitis mouse model, viral RNA loads were assessed at timepoints throughout disease progression. Some mice were viremic at 2 dpi (Fig 6A). CC057 liver viral RNA loads peaked at 3 dpi but viral RNA loads in the liver decreased to below the LOD by 7 dpi before increasing slightly at the 10–11 dpi terminal timepoints. CC057 brain viral RNA loads were dramatically increased at 7 dpi. Brain viral RNA loads were also high when assessed late in the disease course at all timepoints up until euthanasia or death. Interestingly, viral RNA loads did not peak first in the left sciatic nerve as would be expected if RVFV trafficked to the brain via the sciatic nerve following left FP injection, but rather both left and right sciatic nerve viral RNA loads increased after viral RNA was detectable in the brain. Viral RNA was sporadically present in spinal cord sections as early as 3 dpi, with peak spinal cord loads coinciding with peak brain loads at 7 dpi.

CBC and CHEM data supported a biphasic disease course in CC057 mice with mice displaying leukopenia and lymphopenia at 5 dpi and 10–11 dpi (Fig 6B). CC057 mice also exhibited neutrophilia at all timepoints except early at 2 dpi and immediately after liver insult at 5 dpi. CC057 mice had anemia during the acute phase of infection then progressed on to hemoconcentration with accompanying thrombocytosis at the later timepoints during encephalitic disease. Levels of the hepatic indicators of liver damage (ALT) and synthetic function (albumin), also differed significantly from baseline at 5 dpi immediately following the liver infection phase (Fig 6C). CHEM data with no physiologically relevant changes are included in the supplementary material (S6 Fig).

After the initial manifestation of liver cytopathology at 3 dpi, CC057 mice continued to display focal areas of inflammation and necrosis at 5 dpi, although more sparsely distributed (3 of 3 mice) (Fig 6D). CC057 livers had very low levels of antigen staining by 5 dpi (3 of 3 mice). By 7 dpi, the livers of CC057 mice lacked antigen staining altogether (3 of 3 mice) and displayed signs of regenerative activity post-insult including a marked increase in hepatocellular mitotic rate by 7 dpi (3 of 3 mice) and eventual hepatocellular mineralization at 11 dpi (1 of 3 mice). The late-encephalitic phase of infection in CC057 mice was marked by the appearance of necrotic lesions in the brain as early as 7 dpi (1 of 3 mice) and high levels of viral antigen (2 of 3 mice) (Figs 6D and S7). Mouse brains also displayed high levels of viral antigen on 9 dpi (3 of 3 mice) and 11 dpi (2 of 3 mice). Noted brain pathology from 7–11 dpi included patchy to focally extensive acute cortical neuronal necrosis, neuronal dropout, perivascular inflammatory infiltrates, patchy meningeal perivascular cuffing, and infrequent focal meningeal thrombosis. IHC showed an uneven distribution of viral antigen staining throughout the brain at various timepoints starting at 7 dpi (Figs 6D and S7). Antigen was noted in the cortex, the hippocampus, and in deep grey matter regions but was largely lacking in the cerebellum. Additional brain imaging of whole brain sections is included in the supplementary material (S7 Fig).

**Fig 7:**
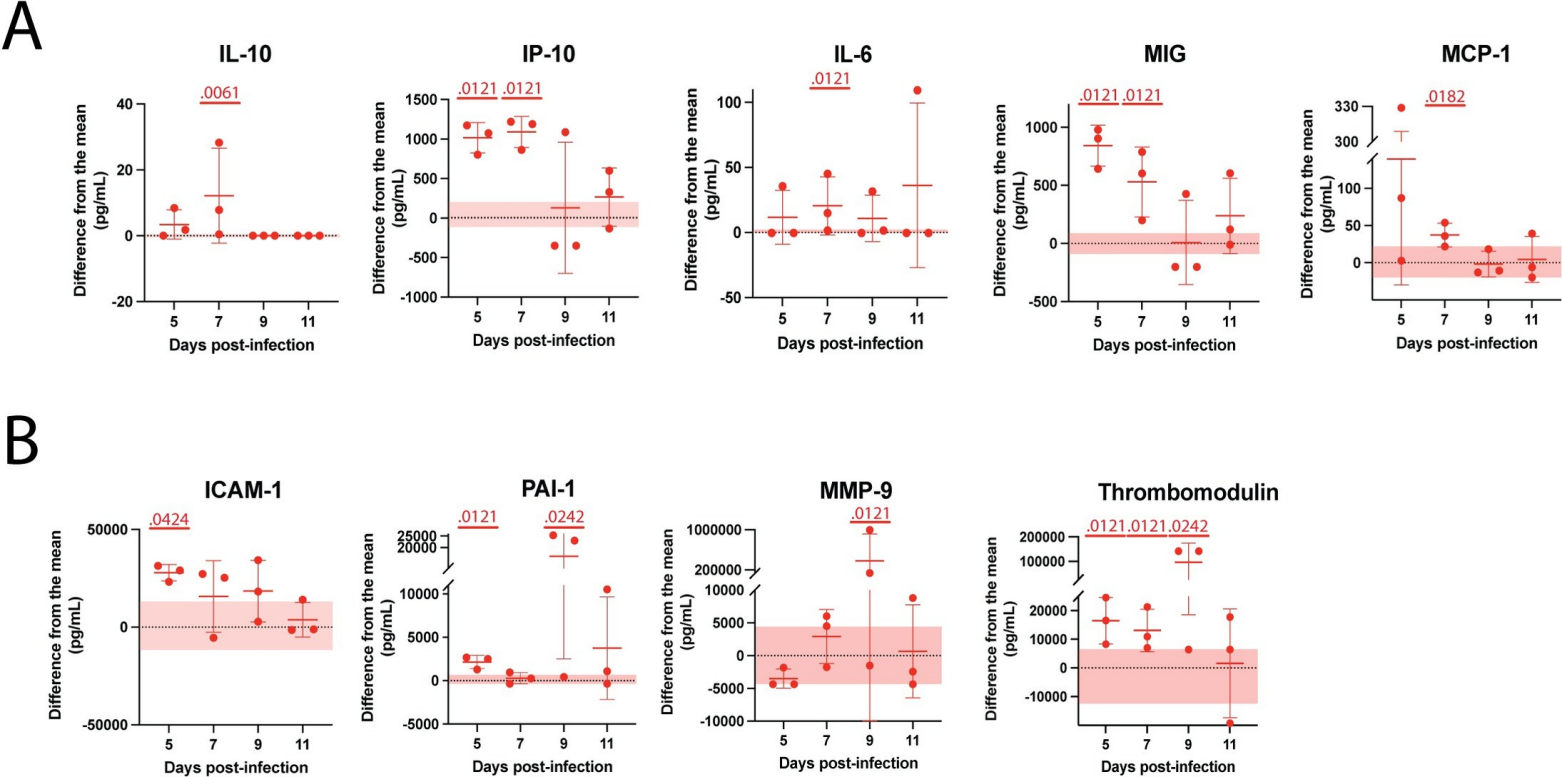
Cytokine signaling and markers of endothelial activation in RVFV-infected CC057 mice. Analyte concentrations in the plasma over time with data presented as a function of difference from the uninfected mean, shown as mean ± SD (n = 3 mice/time point). Each data point shown represents an individual mouse. Pink horizontal shading represents uninfected normal ranges from the mean for CC057 mice. For each analyte, comparisons were performed at each timepoint by Mann-Whitney to compare RVFV-infected samples to uninfected control samples. A) Cytokines and chemokines: IL-10 (7 dpi p = 0.0061); IP-10 (5 dpi p = 0.0121; 7 dpi p = 0.0121); IL-6 (7 dpi p = 0.0121); MIG (5 dpi p = 0.0121; 7 dpi p = 0.0121); MCP-1 (7 dpi p = 0.0182). B) Tissue barriers: ICAM (5 dpi p = 0.0424); PAI-1 (5 dpi p = 0.0121; 9 dpi p = 0.0242); MMP-9 9 dpi (p = 0.0121); Thrombomodulin (5 dpi p = 0.0121; 7 dpi p = 0.0121; 9 dpi p = 0.0242).

To further characterize the later course of disease in CC057 mice, multiplex immune assays were run at various timepoints after hepatic recovery to assess plasma analyte concentrations. Of the 32 assessed analytes, 9 were found to be both statistically significant and deemed to play a physiologically plausible role in clinical disease (Fig 7). Significant elevation in five inflammatory cytokines and chemokines were found between 5 and 7 dpi, peaking concurrent with the time of detection of virus in the central nervous system (CNS) (IL-10, IP-10, IL-6, MIG, MCP-1) (Fig 7A). Of the analytes related to endothelial function, ICAM-1, PAI-1, and thrombomodulin were elevated at 5 dpi prior to detection of virus in the CNS, while PAI-1, MMP-9, and thrombomodulin peaked at 9 dpi after viral CNS infection was well established (Fig 7B). Remaining analyte data are included in the supplementary material (S8 Fig). IFN-α, CD40L, and IL-9 are not included in the supplement due to a lack of data points above the limit of detection.

## Discussion

Classic inbred mice almost uniformly succumb to acute hepatitis following WT RVFV infection [5, 6, 26–28]. BALB/c mice are the exception to this, displaying a split-phenotype where some BALB/c mice overcome the hepatic stage of infection to progress to death by late-onset encephalitis [6]. By using the inherent difference in hepatic disease susceptibility present in BALB/c and MBT mice (classically susceptible to hepatitis) three RVF susceptibility loci (Rvfs2, Rvfs11, and Rvfs5) were identified as affecting survival time [7, 29]. To date, these are the only genetic loci that have been identified as affecting RVFV susceptibility in the mouse. Given the usefulness of murine models for host genetics and immunity studies as well as high-throughput pre-clinical evaluation of vaccines and therapeutics, murine models for other forms of RVF disease are needed. In this study, we utilized the CC resource with the goal of identifying additional RVF disease phenotypes. A comprehensive analysis of RVF disease manifestations in 20 genetically diverse CC strains identified a murine model of late-onset RVF encephalitis. Importantly, this model uses a footpad administration of virus which mimics a peripheral route of exposure, as might be seen during infection via mosquito bite.

This model provides a critical tool for elucidating factors that control viral infection in the liver and provides a tractable murine model in which the route of virus entry into the brain can be dissected. Moreover, future studies using additional CC strains, will permit quantitative trait locus (QTL) mapping of the genetic loci that dictate these divergent clinical outcomes following RVFV infection. However, even without genetic loci mapping, this study illuminates multiple aspects of RVF disease.

### The host factor Mx1 does not confer resistance to RVF disease in mice

All CC strains, regardless of disease phenotype, succumbed to RVFV infection despite all possessing a functional Mx1 locus. Wild-derived, i.e., functional, Mx1 has been associated with increased Influenza A virus resistance in mice and is also known to inhibit its replication [30, 31]. With most inbred mice containing a non-functional Mx1 locus, we hypothesized that CC strains containing wild-derived Mx1 would show increased survival after RVFV challenge [25, 32, 33]. However, the universal lethality of RVFV in the 20 CC strains eliminates Mx1 as the main host genetic factor responsible for mouse susceptibility to RVFV.

### CC mouse phenotypes share similarities to those observed in humans and other animal models

CC mice classified as hepatic presented with gross liver pathology, high liver viral RNA loads, viremia, and elevated ALP, ALT, and bile acids. These disease characteristics match previously established RVF hepatitis models and human cases [4–6, 26–28, 34, 35]. Mixed phenotype CC mouse strains paralleled the disease course in BALB/c mice in that they died at a median of 5–8 dpi, where (within strains) earlier deaths were associated with hepatic disease while later deaths manifested as encephalitis [6]. Encephalitic CC mice presented with CNS symptoms, high viral brain RNA load with clearance of viral RNA from the periphery, and an absence of clinical markers of liver involvement in the later stage of disease. These findings correlated with previously described rat and NHP models of encephalitis and human cases [4, 10, 15–17, 34, 36].

### CC057 mice are resistant to severe hepatitis caused by RVFV

Surprisingly, little to no tissue viral RNA, liver damage, or liver antigen staining were found in either C57BL/6 or CC057 mice until 3 dpi. This signals an extremely rapid progression of disease in C57BL/6 mice for they succumbed consistently between 3–4 dpi with 8–9 logs of viral RNA in their liver. At 3 dpi, unique signatures were seen between C57BL/6 and CC057 models in the levels of viral replication, hepatocellular damage and infection, immune response to infection, and liver dysfunction. The encephalitic CC057 model displayed significantly lower liver viral RNA loads, liver damage, and hepatocellular infection and exhibited self-limiting hepatic disease. C57BL/6 mice were extremely sick at 3 dpi and exhibited common markers of severe viral hepatic infection often seen in fatal human cases including leukopenia, lymphopenia, thrombocytopenia, and elevated ALT and ALP [4, 34, 35]. Contrastingly, CC057 mice displayed a notable absence of severe hematological change and showed only minor elevation in most immunologic analyte concentrations at 3 dpi. This was juxtaposed with the total immunologic dysfunction seen in late-stage disease in C57BL/6 mice and which is characteristic of fatal RVF disease in mice [27]. These distinctive signatures indicate an innate ability to control liver disease in the CC057 model.

### Factors that control RVF hepatitis do not necessarily prevent RVF encephalitis

Despite a self-limiting liver disease course, CC057 mice progressed to CNS invasion and death. This phenomenon of RVF disease progression to the CNS has been documented in other experimental systems but not in a uniform manner following peripheral exposure in mice. [6, 36–38]. In the CC057 model, progression on to late-stage disease was marked by clearance of viral RNA from the liver and viral detection in the CNS by 7 dpi. High viral RNA loads in the brain at 7 dpi and onward were accompanied by cortical lesions and patchy antigen staining throughout the brain parenchyma. In animals challenged by intranasal or aerosol RVFV inoculation, RVFV has been shown to first infect the olfactory bulb then spread caudally into the cerebrum and cerebellum [12, 13, 39]. However, the method of entry into the CNS is unknown for challenge that begins at the periphery such as infection by mosquito bite or FP injection. One possible route of brain entry from an initial peripheral challenge is retrograde neuronal transport, as is known to occur with Rabies virus [40–42]. However, in CC057 mice, viral RNA appeared earliest at very low levels in spinal cord sections at 3 and 5 dpi. At 7 dpi, brain and spinal cord sections peaked with high viral RNA loads while the sciatic nerves remained near the LOD for viral RNA until 9–11 dpi. These data suggest that trafficking via the sciatic nerve is not the mode of viral entry into the CNS for the CC057 encephalitis model, although we cannot rule out that the sciatic nerve was transiently infected and did not get detected due to sampling bias.

### Biomarkers of clinical outcome

Serum viral RNA loads measured at 2 dpi did not correlate with disease outcome or time to death among the CC strains. Although a general trend can be seen between the three disease categorizations, the difference in RNA load is not large and outlier strains such as CC071 and CC058 prevent identifying a direct correlation between serum viral RNA load at 2 dpi and time to death.

Despite the presence of neutralizing antibody levels, no CC mice challenged at the 2 TCID_50_ dose were able to prevent or overcome CNS disease, likely due to the timing of the humoral response. Neutralizing antibody titers against RVFV did not appear in terminal mice until 8 dpi while virus was present at high levels in the brains of CC057 mice by 7 dpi as assessed by qRT-PCR and IHC. It is therefore possible that brain damage is too severe by the time a humoral response is mounted or that antibodies are not able to effectively clear virus from the brain once RVFV has reached the brain parenchyma. Interestingly, 1/3 mice survived infection when challenged at the higher 2,000 or 200,000 TCID_50_ RVFV doses. We hypothesize that challenge at a higher viral dose leads to survival in a minority of mice due to an increased activation of the innate immune response. It is possible that increased innate immune sensing, signaling, and downstream effects lead to more substantial control of viral replication early in the course of infection. A decrease in viral amplification could decrease the likelihood of viral spread to the brain. Alternatively, an increase in innate immune mediators such as IFN, could prime the CNS, making it more resilient against viral infection thus resulting in survival in some mice. Further study will be crucial to understand the mechanism of survival at higher viral challenge doses in the CC057 model.

A prominent feature of RVF disease in CC057 mice was a sustained neutrophilia from 3 dpi to the point of death with the only drop occurring at 5 dpi. The decrease in circulating neutrophils at 5 dpi could be due to liver infiltration during the hepatic disease phase. During the end stage of disease, CC057 mice present with significant leukopenia and lymphopenia suggestive of brain infiltration or cellular death. As has been seen in other animal models of RVF encephalitis, increases in cytokine, chemokine, and endothelial-related blood markers were seen in the CC057 model late in the course of disease [36, 37]. Important increases in inflammatory and chemoattractant markers were seen in IP-10, IL-6, MIG, and MCP-1 with an accompanying increase in the anti-inflammatory cytokine IL-10. All five of these markers have been shown to be elevated in human RVF cases [35, 43]. IL-6 and MCP-1 elevation has also been shown in lethal intranasally infected mice [13]. Intriguingly MIG (monokine induced by IFN-γ) was the most elevated at 5 dpi, two days before viral entry into the brain, indicating active IFN-γ signaling prior to the time virus was detected in the brain. There is also evidence of T-cell and monocyte recruitment at 5 and 7 dpi due to elevation of IP-10 and MCP-1 respectively, at these timepoints. However, these increases in cytokine and chemokine production are likely too late in the course of disease to offer mice protection and perhaps are harbingers of leukocyte trafficking that could facilitate viral entry into the brain.

While the significance of analyte increases in this model are not yet completely understood, we speculate that they could signal brain endothelial cell infection or breach of the CNS by RVFV. ICAM-1 peaked the earliest of the endothelial-related markers at 5 dpi in the blood, signaling it has been shed by an activated endothelium. This increase in ICAM-1 before detectable virus in the brain could serve as an early biomarker of CNS invasion. The lack of overwhelming increases in measured blood inflammatory markers before virus is detected in the brain at 7 dpi suggests that RVFV gains entry to the brain without the need for BBB breakdown. Peaks in markers signaling potential BBB breakdown such as Total PAI-1, MMP-9, and thrombomodulin do not occur until 9 dpi during the endpoint of disease [44]. These findings are supported by work in various animal models detailing that BBB breakdown is not required for RVFV entry into the brain and often only occurs late in the course of disease after the virus has already caused severe brain damage [13, 45]. Further study into the route of viral brain invasion upon peripheral infection is an essential next step and can be accomplished by using the novel CC057 mouse model.

## Conclusion

In conclusion, we describe here a comprehensive analysis of RVF disease manifestations in 20 CC mouse strains resulting in the characterization of three phenotypes: hepatitis, mixed phenotype, and encephalitis. Large differences in viral load kinetics, pathologies, CBC, CHEM, and blood analytes were found between hepatic and encephalitic clinical outcomes. Of the challenged CC strains, the CC057 strain was identified as a murine model of uniform late-onset RVF encephalitis, and a detailed analysis of phenotype was performed by virologic, pathologic, hematologic, histologic, and immunologic assessment. Our data suggest that host factors play a critical role in determining RVF disease manifestations and we demonstrate that the genetic diversity provided by the CC resource enables the identification of novel experimental systems for the study of RVF disease manifestations. The CC resource allows us to link identified outcome to host genotype, therefore future work will focus on identifying genes associated with protection or susceptibility from various RVF clinical outcomes. The CC057 model described in this paper will enable immediate investigation into the pathogenesis of RVF CNS disease, identification of the genetic basis for disease variation, and evaluation of therapeutic strategies that have direct implications for treatment of RVF encephalitis.

## Materials and methods

### Ethics statement and biosafety information

Animal research was approved by University of Pittsburgh Institutional Animal Care and Use Committee (IACUC) (protocols 19044158 and 22030821). All experiments with the WT RVFV ZH501 strain were performed in the University of Pittsburgh regional biocontainment biosafety level 3 laboratory.

### Virus generation, growth, and titer

Pathogenic rRVFV was generated using reverse genetics based on the ZH501 strain background [46–48]. Virus stocks were grown to passage 2 and fully sequence confirmed using next-generation sequencing prior to use. Viral stock titers were determined by 50% tissue culture infective dose (TCID50) assay as described previously [5, 49]

### Collaborative cross mouse strain screening

All Collaborative Cross mice used in this study were obtained from the Systems Genetics Core Facility at the University of North Carolina [50]. Previous to their relocation to UNC, CC lines were generated and bred at Tel Aviv University in Israel [51], Geniad in Australia [52] and Oak Ridge National Laboratory in the US [53]. Mice used in this study: 4- to 12-week-old female CC001/Unc, CC005/TauUnc, CC011/Unc, CC013/GeniUnc, CC023/GeniUnc, CC027/GeniUnc, CC028/GeniUnc, CC030/GeniUnc, CC031/GeniUnc, CC035/Unc, CC039/Unc, CC042/GeniUnc, CC044/Unc, CC058/Unc, CC060/Unc, CC062/Unc, CC068/TauUnc, CC071/TauUnc, CC078/TauUnc, and female and male CC057/Unc. All mice were housed in HEPA filtration racks with ad libitum access to food and water. Mice were infected with 2–200,000 TCID_50_ recombinant WT RVFV ZH501 strain diluted in phosphate buffered saline (PBS) under isoflurane anesthesia via left rear FP injection to model a mosquito bite. For all experiments, mice were weighed and evaluated daily for clinical signs of disease and euthanized according to a predetermined clinical scoring method as previously described [5]. At 2 dpi, blood was collected via lateral saphenous bleed for quantification of viral RNA. At the time of euthanasia, mice were anesthetized with isoflurane and blood was collected via cardiac puncture for qRT-PCR, ELISA, FRNT, CBC, and CHEM. CBC and CHEM data were analyzed using a VETSCAN HM5 hematology analyzer (Abaxis) and a VETSCAN VS2 chemistry analyzer (Abaxis) using the Mammalian Liver Profile reagent rotor, respectively. Following cervical dislocation, liver, spleen, kidney, small intestine, eye, and brain were collected in PBS supplemented with antibiotics and antimycotic (Invitrogen) and homogenized as previously described [5].

### Serial euthanasia of C57BL/6 and CC057/Unc mice

4- to 12-week-old female and male C57BL/6J (stock #000664) mice, purchased from Jackson Laboratories, and 4- to 12-week-old female and male CC057/Unc mice were infected with 2 TCID_50_ recombinant WT RVFV ZH501 strain as above or mock-infected with PBS to serve as negative controls. C57BL/6J mice were euthanized at 0.5, 1, 2, and 3 dpi. CC057/Unc mice were euthanized at 0.5, 1, 2, 3, 5, 7, 9, 10, and 11 dpi. At each timepoint, blood, tissue samples, and whole organs were collected from both infected and mock-infected animals. Blood was taken for qRT-PCR, CBC, CHEM, and multiplex assays. Tissue samples were taken for quantification of viral RNA in order (liver, spleen, right sciatic nerve, left sciatic nerve, upper spinal cord section, middle spinal cord section, lower spinal cord section, and brain) with instruments cleaned in ethanol between each harvest. Whole organ tissue sampling order: liver, spleen, and brain whole organs were collected and fixed in 10% formalin for pathological analysis.

### RNA extraction and quantitative RT-PCR

RNA was extracted from homogenized tissue samples with TRIzol reagent, and quantitative reverse transcription-PCR (qRT-PCR) targeting the L segment of RVFV [54] was performed [5]. RNA copies for each unknown sample were determined by comparison to a standard L RNA curve and normalized by tissue weight or serum volume. Assay LODs are reported on all graphs at 473 RNA copies for tissue samples, 9,480 RNA copies for terminal plasma samples, and 18,960 RNA copies for bleed serum sample. Tissue LOD was calculated as the highest threshold cycle (CT) value detected in the standard curve multiplied by 100, to account for dilutions, and divided by the average sampled tissue weight. Blood LODs were calculated as the highest threshold cycle (CT) value detected in the standard curve multiplied by 200 (terminal plasma) or 400 (bleed serum), to account for dilutions.

### Enzyme-linked immunosorbent assay

ELISAs were performed as described previously [5] using plates coated with RVFV-infected or uninfected Vero E6 cell lysate. Endpoint ELISA titers were defined as the highest dilution of plasma that resulted in an OD value at least two standard deviations above the average obtained from all negative mouse plasma control wells.

### Focus reduction neutralization assay

Mouse plasma was serially diluted, in duplicate, and incubated with 200 focus-forming units of DelNSs/DelNSm RVFV as described previously [55]. Foci were detected using Moss TMB-H peroxidase substrate (MossBio) and counted using an immunospot reader (CTL). Percent neutralization was calculated by comparing sample wells to wells containing virus but no plasma. The dilution of plasma at which 50% of foci were neutralized is reported as FRNT_50_.

### Histopathology

Fixed liver, spleen, and brain tissues were processed, paraffin embedded, and sectioned using standard methods. Tissues were stained with hematoxylin and eosin (H&E) for visualization. IHC assays were performed through the Pitt Biospecimen Core. Tissues were evaluated for anti-RVFV immunoreactivity using a polyclonal rabbit anti-N protein antibody (1:200, Genscript, custom). Appropriate negative control tissues were included at each timepoint for each mouse strain.

### Multiplex assays

Plasma samples collected during the serial euthanasia experiment, from both mock-infected and RVFV-infected mice, were analyzed using commercial multiplex assays according to the manufacturer’s instructions. 32 analytes were assessed in 8 commercially available assays. Analyte levels measured in 8 uninfected CC057 mice and 8 uninfected C57BL/6 mice were used to calculate normal ranges for each analyte. Millipore assays: thirteen-plex assay for keratinocyte chemoattractant (KC), monokine induced by gamma interferon (MIG, CXCL-9), monocyte chemoattractant protein 1 (MCP-1, CCL2), interferon gamma-induced protein 10 (IP-10, CXCL-10), interleukin 1 alpha (IL-1α), interleukin 10 (IL-10), regulated on activation normal T-cell-expressed and secreted (RANTES, CCL-5), interleukin 6 (IL-6), tumor necrosis factor alpha (TNFα), macrophage inflammatory protein-1 alpha (MIP-1α, CCL3), macrophage inflammatory protein-1 beta (MIP-1β, CCL4), interferon gamma (IFN-γ), and interleukin 9 (IL-9); seven-plex assay for E-Selectin, intercellular adhesion molecule (ICAM), platelet endothelial cell adhesion molecule (Pecam-1), P-Selectin, plasminogen activator inhibitor-1 (PAI-1), matrix metallopeptidase 9 (MMP-9), and Thrombomodulin; four-plex assay for interferon beta (IFN-β), Fractalkine, macrophage-derived chemokine (MDC, CCL22), and macrophage inflammatory protein-3 (MIP-3β, CCL19); two-plex assay for angiopoietin-2 (Ang-2) and hepatocyte growth factor (HGF); two-plex assay for tumor necrosis factor receptor I (TNFRI) and tumor necrosis factor receptor II (TNFRII); two-plex assay for interleukin 28 (IL-28B) and CD40L. ThermoFisher assays: single-plex assays for C-reactive protein (CRP) and interferon alpha (IFN-α). Data were collected on a Bio-Plex 200 (Bio-Rad) instrument. All assay results were reported either as raw data or as the difference from the mean of mouse strain-specific mock infected samples (shown as either pg/mL or mg/dL).

### Statistical analysis

Data were entered into GraphPad Prism 9 for statistical analysis and graphing. qRT-PCR data were analyzed in Excel. Specific statistical tests for each data set are indicated in the figure legends.

## Supporting information

S1 Fig Viral RNA loads across CC strains in other tissues in RVFV-infected mice. qRT-PCR based assessment of viral RNA loads in tissues at time of euthanasia. CC strains are separated into three categories of disease by two vertical dashed grey lines. Data shown as geometric mean ± geometric SD. LOD of assay noted by dotted horizontal line.
(TIF)

S2 Fig CHEM and CBC profiles across CC strains after RVFV infection. CC strains are separated into three categories of disease by two vertical dashed grey lines. A) CHEM and B) CBC analysis was run if sufficient sample was present (n≤5 per CC strain). Data shown as mean ± SD. If data were outside the LOD, the upper or lower LOD for assays are noted by horizontal dotted line. Blood Urea Nitrogen upper LOD: 180 mg/dL; Cholesterol lower LOD: 20 mg/dL.
(TIF)

S3 Fig Virus-specific antibodies develop too late in infection to prevent RVFV spread to the CNS. (A) ELISA and (B) FRNT of plasma at time of euthanasia following RVFV challenge [n = 5/CC strain, except CC057 n = 9 (5 female and 4 male)]. Upper and lower LODs for each assay noted by dotted lines. ELISA upper LOD: 218,700; ELISA lower LOD: 100; FRNT_50_ upper LOD: 2,560; FRNT_50_ lower LOD: 20.
(TIF)

S4 Fig Additional CHEM data in RVFV-infected C57BL/6 and CC057 mice. Data presented as a function of difference from the uninfected mean, shown as mean ± SD (N = 3-7/time point). The uninfected means and normal ranges for C57BL/6 and CC057 mice were determined by performing CHEM on blood from 9 uninfected C57BL/6 mice and 9 uninfected CC057 mice. Uninfected normal ranges from the mean for C57BL/6 and CC057 mice are represented by grey and pink horizontal bars respectively. Comparisons of CHEM data were performed at each timepoint by Mann-Whitney to compare RVFV-infected samples to uninfected control samples for each mouse strain separately.
(TIF)

S5 Fig Baseline inflammatory environment is lower in CC057 mice than C57BL/6 mice. Analyte concentrations in uninfected C57BL/6 and CC057 mice. Data shown as mean ± SD (n = 8/strain). Comparison of baselines for each analyte was performed by Mann-Whitney (E-Selectin p = 0.0045; ICAM-1 p = 0.0104; MMP-9 p = 0.0002; Thrombomodulin p = 0.003; TNFRI p = 0.0002; TNFRII p = 0.0002; MCP-1 p = 0.0065; MIP-1β p = 0.0126; Fractalkine p = 0.0002; MDC p = 0.0104; MIP-3β p = 0.0134). Lower LOD for assays noted by horizontal dotted line. E-Selectin LOD: 1000pg/mL; ICAM-1 LOD: 200pg/mL; MMP-9 LOD: 1000pg/mL; Thrombomodulin LOD: 8200pg/mL; TNFRI LOD: 450.95pg/mL; TNFRII LOD: 62pg/mL; MCP-1 LOD: 6.4pg/mL; MIP-1β LOD: 37.32pg/mL; Fractalkine LOD: 48.48pg/mL; MDC LOD: 4.74pg/mL; MIP-3β LOD: 29.96pg/mL; CRP LOD: 0.000004mg/dL; IFN-α LOD: 26.72pg/mL; Pecam-1 LOD: 600pg/mL; P-Selectin LOD: 8800pg/mL; PAI-1 LOD: 200pg/mL; KC LOD: 6.96pg/mL; MIG LOD: 27.3pg/mL; IP-10 LOD: 6.26pg/mL; IL-1α LOD: 28.88pg/mL; IL-10 LOD: 27.14pg/mL; RANTES LOD: 6.1pg/mL; IL-6 LOD: 6.42pg/mL; TNFα LOD: 6.56pg/mL; MIP-1α LOD: 161.08pg/mL; IFN-γ LOD: 6.46pg/mL; IL-9 LOD: 37.82pg/mL; IFNβ LOD: 20.18pg/mL; Ang2 LOD: 59.3pg/mL; HGF LOD: 273.68pg/mL; IL-28B LOD: 145.39pg/mL; CD40L LOD: 48.83pg/mL.
(TIF)

S6 Fig Additional CHEM data over time in RVFV-infected CC057 mice. Data presented as a function of difference from the uninfected mean, shown as mean ± SD (N = 3-23/time point). Pink horizontal bars represent uninfected normal ranges from the mean for CC057 mice. The uninfected means and normal ranges for CC057 mice were determined by performing CHEM on blood from 9 uninfected CC057 mice. Comparisons of CHEM data were performed at each timepoint by Mann-Whitney to compare RVFV-infected samples to uninfected control samples for each mouse strain separately (Blood Urea Nitrogen 10–12 dpi p = 0.0192).
(TIF)

S7 Fig Whole brain sections identify RVFV antigen staining in various regions of the brain over time. IHC of RVFV antigen (brown) in formalin-fixed paraffin-embedded mock- or RVFV-infected CC057 brains at different times post-infection. Each brain image represents an individual mouse.
(TIF)

S8 Fig Additional CC057 analyte concentrations in the plasma over time in RVFV-infected mice. Data presented as a function of difference from the uninfected mean, shown as mean ± SD (N = 3/time point). Pink horizontal bars represent uninfected normal ranges from the mean for CC057 mice. For each analyte, comparisons were performed at each timepoint by Mann-Whitney to compare RVFV-infected samples to uninfected control samples. CRP (5 dpi p = 0.0424); MIP-3β (7 dpi p = 0.0121; 11 dpi p = 0.0121); HGF (5 dpi p = 0.0121; 9 dpi p = 0.0121); Fractalkine (7 dpi p = 0.0121; 9 dpi p = 0.0121; 11 dpi p = 0.0424); MDC (9 dpi p = 0.0424); TNFRII (5 dpi p = 0.0121; 9 dpi p = 0.0121; 11 dpi p = 0.0242).
(TIF)
