# Supplementary figures and images for: Genetic diversity of collaborative cross mice enables identification of novel rift valley fever virus encephalitis model
Source: PLoS Pathog. 2022 Jul 14;18(7):e1010649. doi: 10.1371/journal.ppat.1010649 (PMC9282606; doi:10.1371/journal.ppat.1010649)

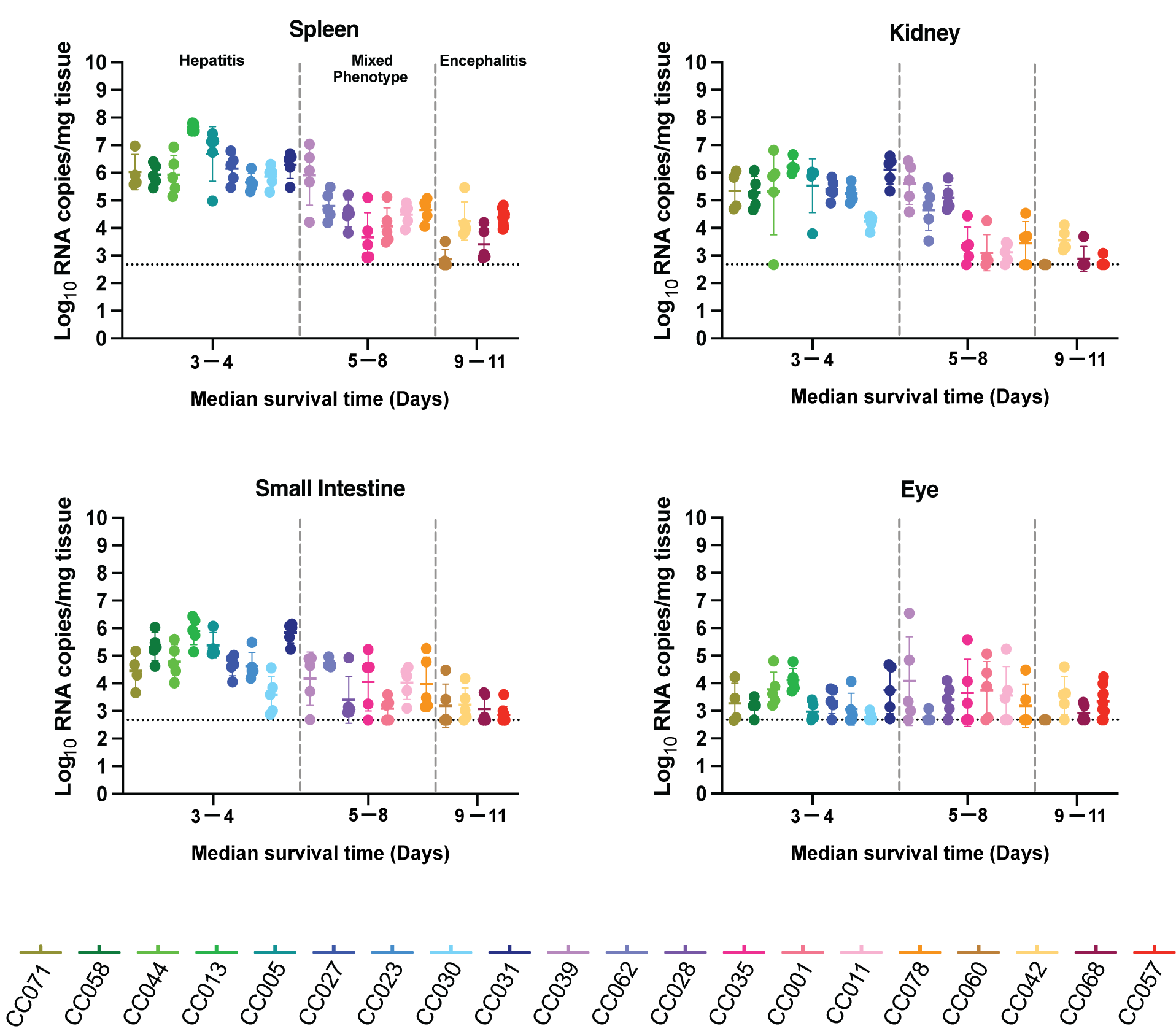

Supplement: S1 Fig — qRT-PCR based assessment of viral RNA loads in tissues at time of euthanasia. CC strains are separated into three categories of disease by two vertical dashed grey lines. Data shown as geometric mean ± geometric SD. LOD of assay noted by dotted horizontal line. (TIF) [file ppat.1010649.s001.tif]

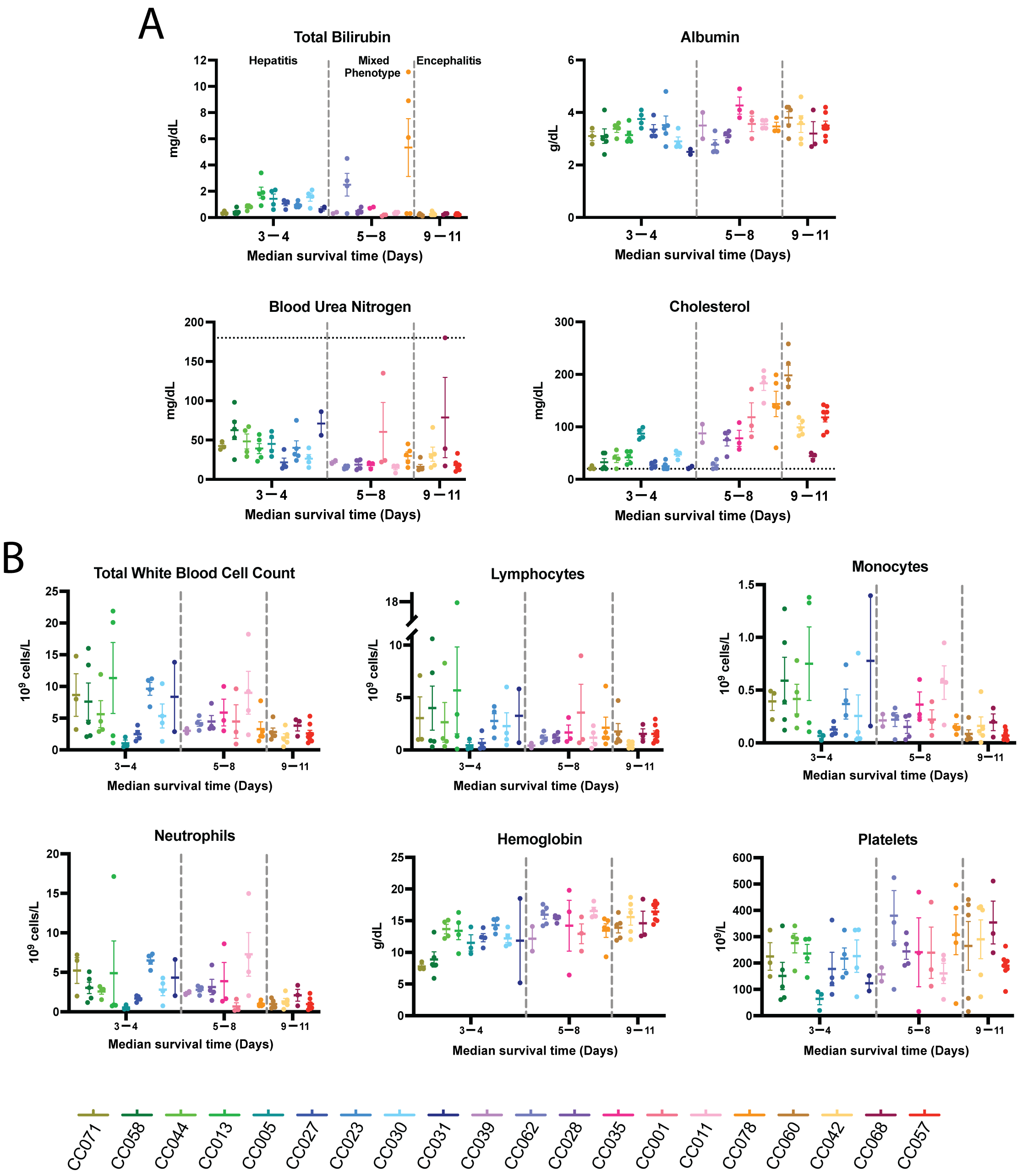

Supplement: S2 Fig — CC strains are separated into three categories of disease by two vertical dashed grey lines. A) CHEM and B) CBC analysis was run if sufficient sample was present (n≤5 per CC strain). Data shown as mean ± SD. If data were outside the LOD, the upper or lower LOD for assays are noted by horizontal dotted line. Blood Urea Nitrogen upper LOD: 180 mg/dL; Cholesterol lower LOD: 20 mg/dL. (TIF) [file ppat.1010649.s002.tif]

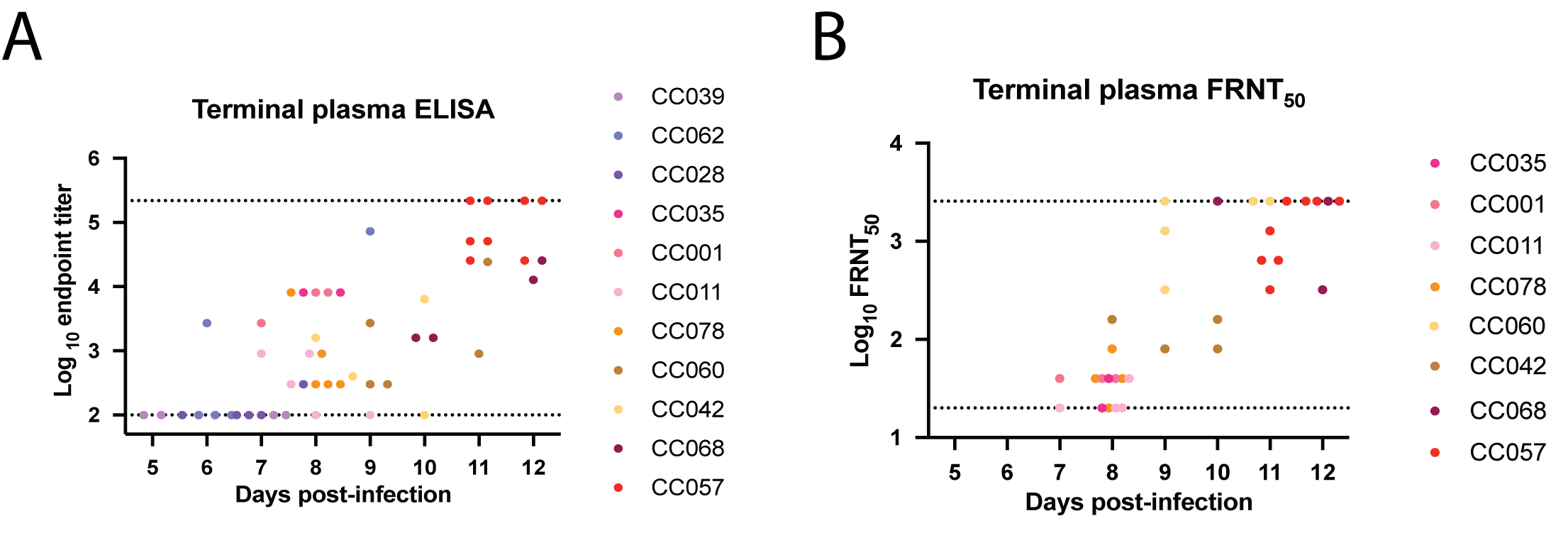

Supplement: S3 Fig — (A) ELISA and (B) FRNT of plasma at time of euthanasia following RVFV challenge [n = 5/CC strain, except CC057 n = 9 (5 female and 4 male)]. Upper and lower LODs for each assay noted by dotted lines. ELISA upper LOD: 218,700; ELISA lower LOD: 100; FRNT50 upper LOD: 2,560; FRNT50 lower LOD: 20. (TIF) [file ppat.1010649.s003.tif]

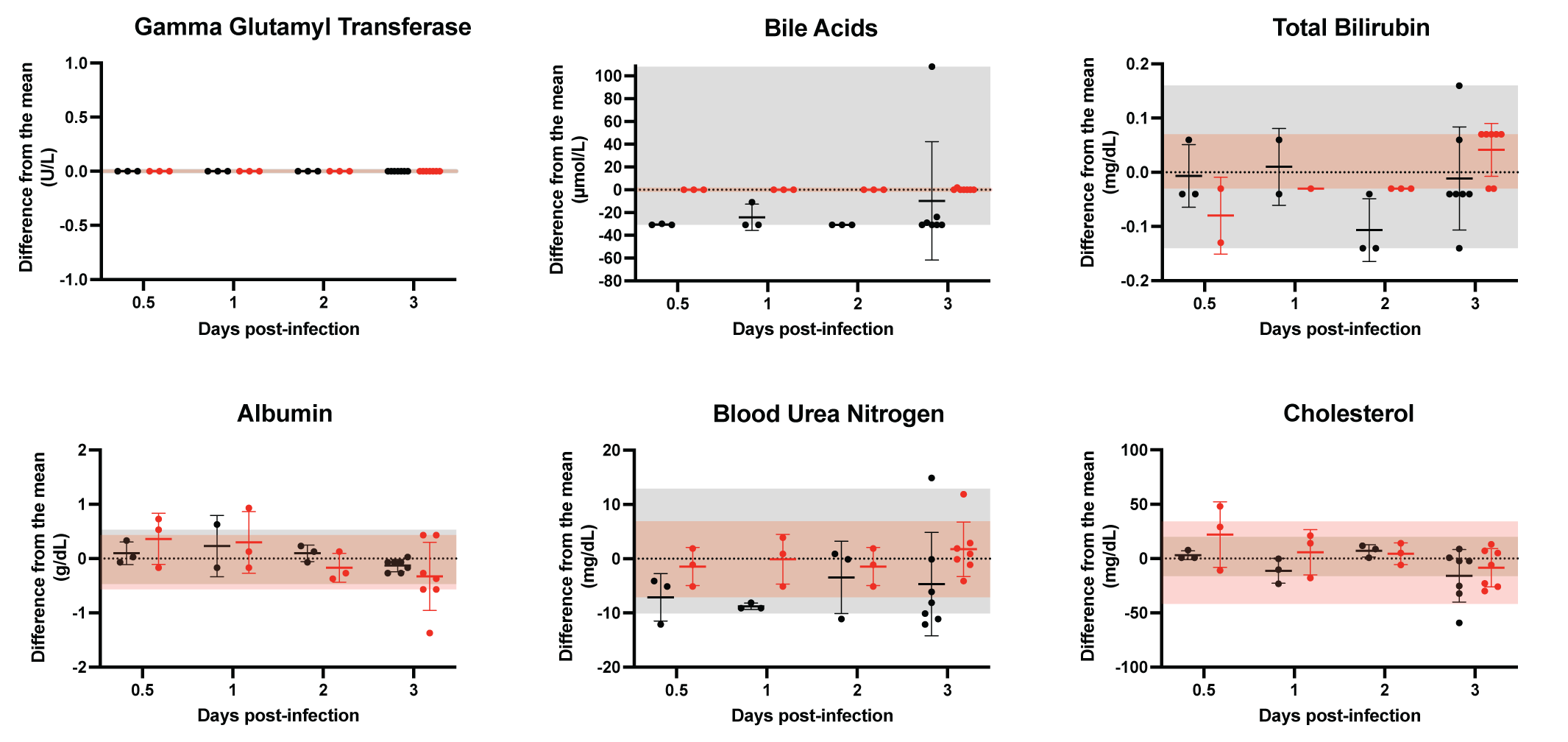

Supplement: S4 Fig — Data presented as a function of difference from the uninfected mean, shown as mean ± SD (N = 3-7/time point). The uninfected means and normal ranges for C57BL/6 and CC057 mice were determined by performing CHEM on blood from 9 uninfected C57BL/6 mice and 9 uninfected CC057 mice. Uninfected normal ranges from the mean for C57BL/6 and CC057 mice are represented by grey and pink horizontal bars respectively. Comparisons of CHEM data were performed at each timepoint by Mann-Whitney to compare RVFV-infected samples to uninfected control samples for each mouse strain separately. (TIF) [file ppat.1010649.s004.tif]

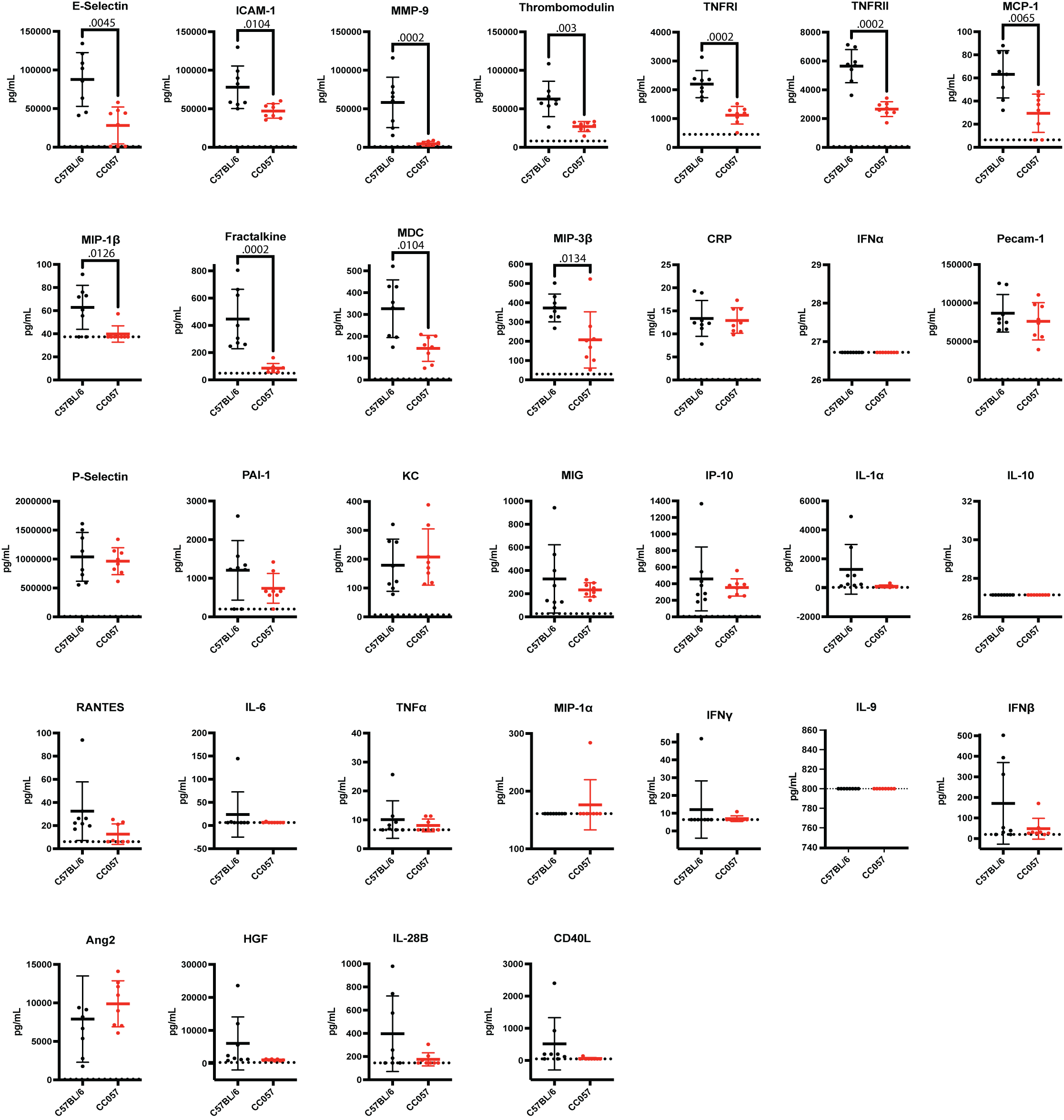

Supplement: S5 Fig — Analyte concentrations in uninfected C57BL/6 and CC057 mice. Data shown as mean ± SD (n = 8/strain). Comparison of baselines for each analyte was performed by Mann-Whitney (E-Selectin p = 0.0045; ICAM-1 p = 0.0104; MMP-9 p = 0.0002; Thrombomodulin p = 0.003; TNFRI p = 0.0002; TNFRII p = 0.0002; MCP-1 p = 0.0065; MIP-1β p = 0.0126; Fractalkine p = 0.0002; MDC p = 0.0104; MIP-3β p = 0.0134). Lower LOD for assays noted by horizontal dotted line. E-Selectin LOD: 1000pg/mL; ICAM-1 LOD: 200pg/mL; MMP-9 LOD: 1000pg/mL; Thrombomodulin LOD: 8200pg/mL; TNFRI LOD: 450.95pg/mL; TNFRII LOD: 62pg/mL; MCP-1 LOD: 6.4pg/mL; MIP-1β LOD: 37.32pg/mL; Fractalkine LOD: 48.48pg/mL; MDC LOD: 4.74pg/mL; MIP-3β LOD: 29.96pg/mL; CRP LOD: 0.000004mg/dL; IFN-α LOD: 26.72pg/mL; Pecam-1 LOD: 600pg/mL; P-Selectin LOD: 8800pg/mL; PAI-1 LOD: 200pg/mL; KC LOD: 6.96pg/mL; MIG LOD: 27.3pg/mL; IP-10 LOD: 6.26pg/mL; IL-1α LOD: 28.88pg/mL; IL-10 LOD: 27.14pg/mL; RANTES LOD: 6.1pg/mL; IL-6 LOD: 6.42pg/mL; TNFα LOD: 6.56pg/mL; MIP-1α LOD: 161.08pg/mL; IFN-γ LOD: 6.46pg/mL; IL-9 LOD: 37.82pg/mL; IFNβ LOD: 20.18pg/mL; Ang2 LOD: 59.3pg/mL; HGF LOD: 273.68pg/mL; IL-28B LOD: 145.39pg/mL; CD40L LOD: 48.83pg/mL. (TIF) [file ppat.1010649.s005.tif]

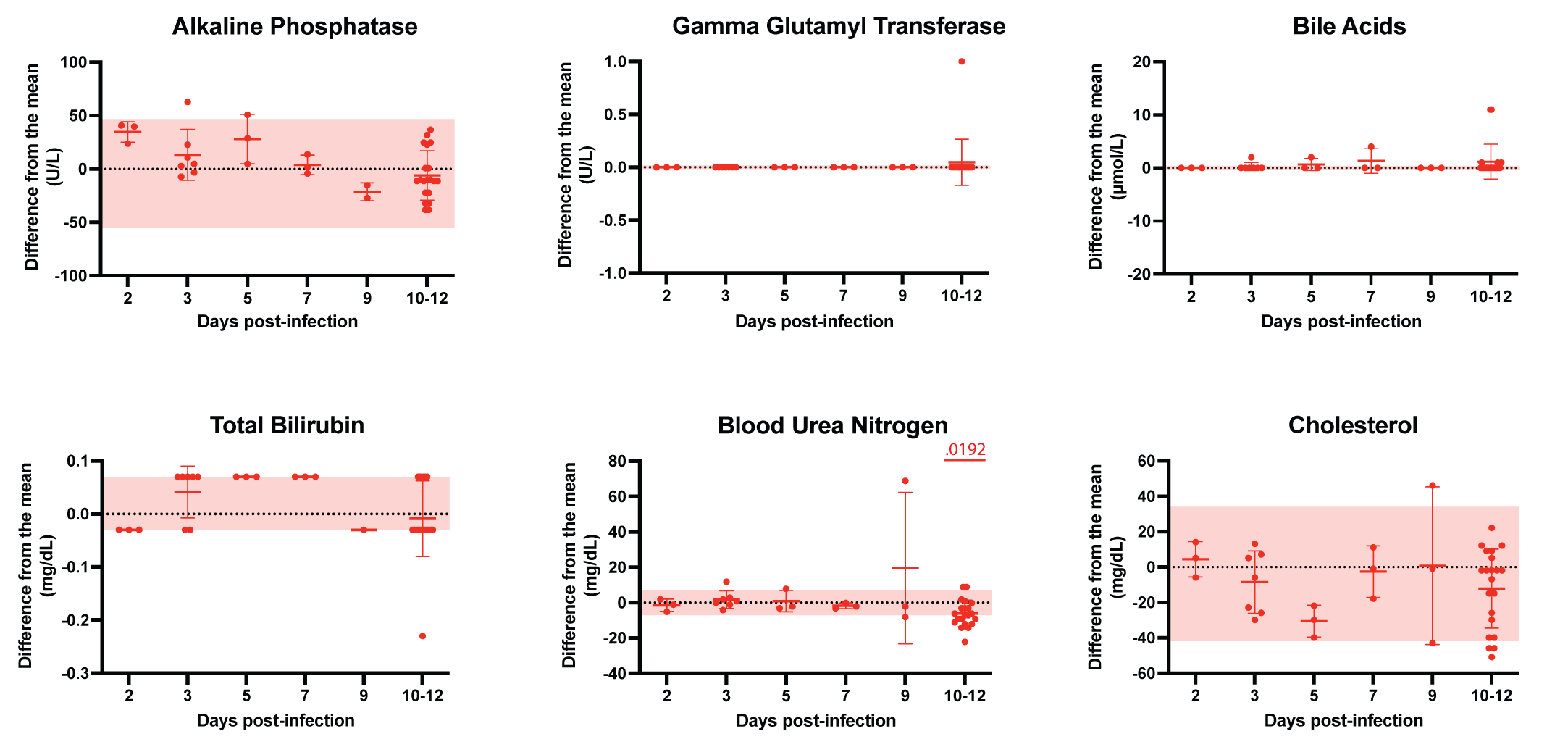

Supplement: S6 Fig — Data presented as a function of difference from the uninfected mean, shown as mean ± SD (N = 3-23/time point). Pink horizontal bars represent uninfected normal ranges from the mean for CC057 mice. The uninfected means and normal ranges for CC057 mice were determined by performing CHEM on blood from 9 uninfected CC057 mice. Comparisons of CHEM data were performed at each timepoint by Mann-Whitney to compare RVFV-infected samples to uninfected control samples for each mouse strain separately (Blood Urea Nitrogen 10–12 dpi p = 0.0192). (TIF) [file ppat.1010649.s006.tif]

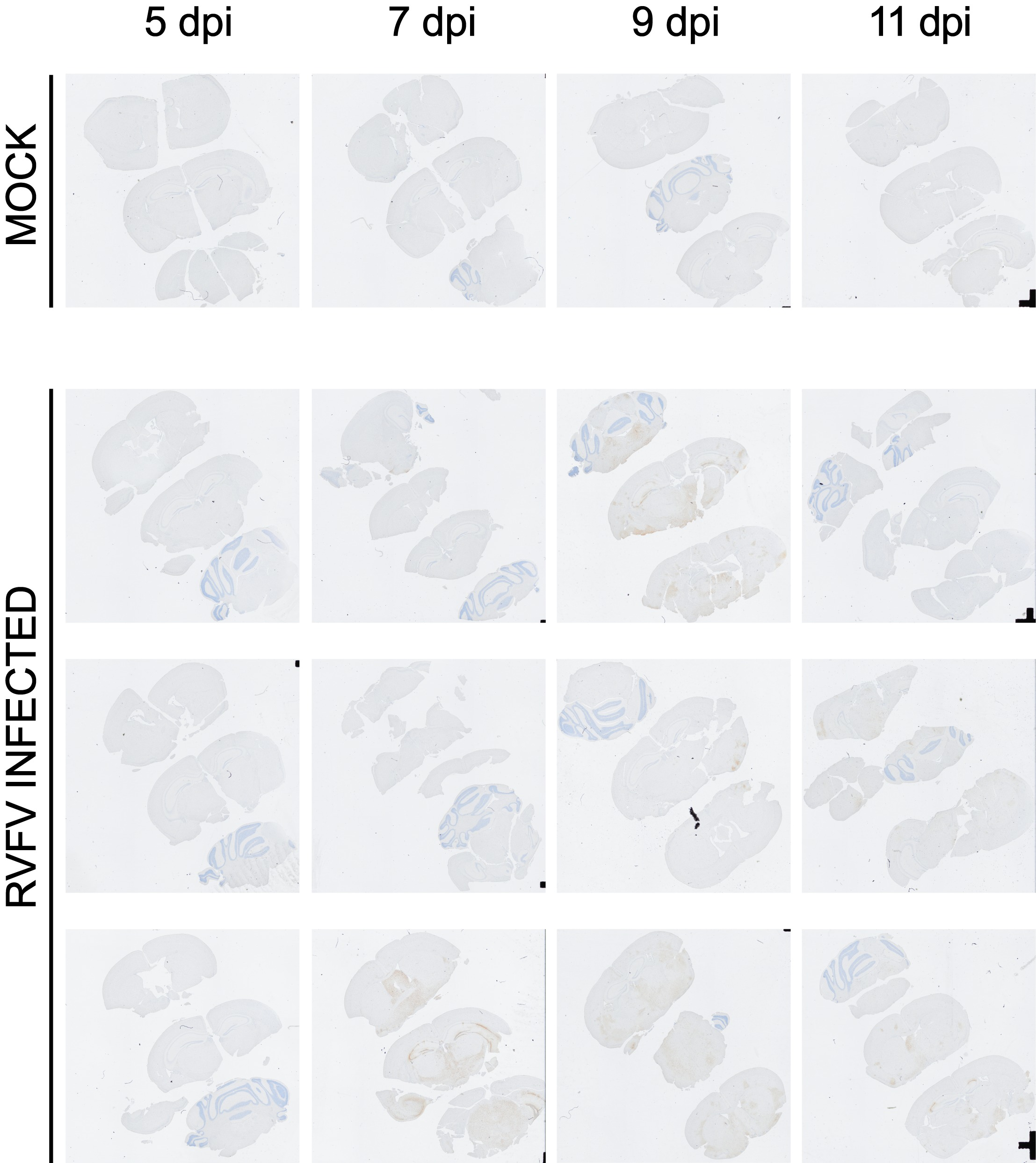

Supplement: S7 Fig — IHC of RVFV antigen (brown) in formalin-fixed paraffin-embedded mock- or RVFV-infected CC057 brains at different times post-infection. Each brain image represents an individual mouse. (TIF) [file ppat.1010649.s007.tif]

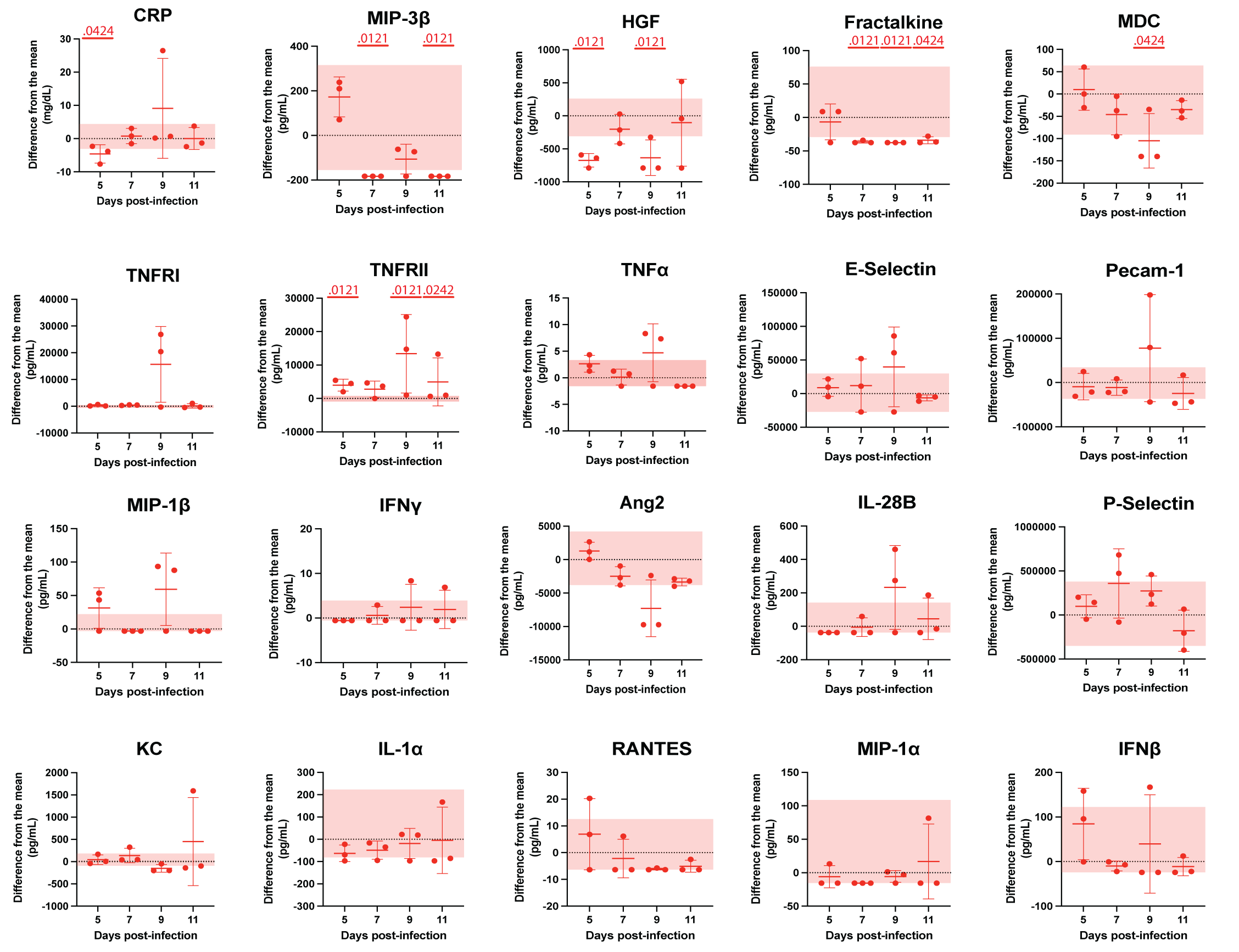

Supplement: S8 Fig — Data presented as a function of difference from the uninfected mean, shown as mean ± SD (N = 3/time point). Pink horizontal bars represent uninfected normal ranges from the mean for CC057 mice. For each analyte, comparisons were performed at each timepoint by Mann-Whitney to compare RVFV-infected samples to uninfected control samples. CRP (5 dpi p = 0.0424); MIP-3β (7 dpi p = 0.0121; 11 dpi p = 0.0121); HGF (5 dpi p = 0.0121; 9 dpi p = 0.0121); Fractalkine (7 dpi p = 0.0121; 9 dpi p = 0.0121; 11 dpi p = 0.0424); MDC (9 dpi p = 0.0424); TNFRII (5 dpi p = 0.0121; 9 dpi p = 0.0121; 11 dpi p = 0.0242). (TIF) [file ppat.1010649.s008.tif]
